# What does a modified-Fibonacci dose-escalation actually correspond to?

**DOI:** 10.1186/1471-2288-12-103

**Published:** 2012-07-23

**Authors:** Nicolas Penel, Andrew Kramar

**Affiliations:** 1 Methodology and Biostatistics Unit, Centre Oscar Lambret, 3 rue Frederic Combemale, 59020, Lille cedex, France; 2 Research Unit EA2694, Medical School, Lille-Nord-de-France University, 1 place de Verdun, 59000, Lille, France

**Keywords:** Dose escalation, Dose increments, Fibonacci sequence, Phase I trials, Methodology

## Abstract

**Background:**

In most phase I oncology trials, it is often stated that the dose increments follow a “modified-Fibonacci sequence”. This term, however, is vague.

**Methods:**

To better characterize this sequence, we reviewed 81 phase I trials based on this concept.

**Results:**

Out of 198 phase I oncology trials, 81 (41%) are based on modified-Fibonacci series. Actual incremental ratios varied in a large range from 0.80 to 2.08. The median of actual increments was about 2.00, 1.50, 1.33, 1.33, 1.33, 1.33, 1.30, 1.35…. The “modified Fibonacci-sequence” gathers heterogeneous variation of the genuine sequence, which does not tend to a constant number at higher dose-levels.

**Conclusion:**

This confusing term should be avoided.

## Background

Dose-finding phase I trials seek to determine an optimal recommended dose for a new compound for further testing in phase II trials [1,2]. For cytotoxic drugs, this dose corresponds to the highest dose associated with an acceptable level of toxicity, referred to as the maximal tolerated dose (MTD). Such trials consist in a design where successive cohorts of patients are treated with increasing doses of the drug. The phase II recommended dose is usually taken as the dose level just below the MTD [1,2]. Endpoints other than the MTD have been considered for molecular targeted therapies such as optimal biological dose (OBD). Nevertheless, two different notions are largely confounded: guidance for determining the actual dose levels to be explored in the trial and guidance for the dose-escalation strategy between dose levels, that is, the determination of the number of patients enrolled at each dose level. Basically there are two approaches for guiding the number of patients enrolled at each dose level: algorithm-based methods such as the classical 3 + 3 and more recent model-based methods such as the different versions of the continual reassessment method [1-3]. The guidance for determining which dose levels are to be explored is usually based on pre-clinical and pharmacokinetic data. Once a starting dose level has been determined, a Fibonacci sequence, or its modified version, is one of the most frequently used methods for determining dose increments.

The genuine and the modified Fibonacci sequence determine dose steps (increments). The genuine Fibonacci sequence is defined by the linear recurrence equation ${\text{F}}_{n}={\text{F}}_{n-1}+{\text{F}}_{n-2}$, which goes like this: 1, 2, 3, 5, 8, 13, 21, 34, 55, 89… In other words, the next number in the sequence is equal to the sum of its two predecessors. The ratio of successive Fibonacci numbers F_n_/F_*n-1*_ (incremental ratio) tends rapidly to a constant ((1 + √5)/2) ≈ 1.61) as *n* approaches infinity. For the purpose of a dose-seeking phase I trial, this sequence offers very appealing guidance for the increment set up; because the absolute doses and the absolute dose change grow larger and larger with constant incremental ratios (≈1.61; ≈+61%) [1,4,5]. The genuine Fibonacci sequence in itself is not commonly used as guidance for establishing dose increments in the trial. Phase I methodologists refer commonly to the “so-called modified Fibonacci sequence” [5], which has the followng incremental ratios: 2.00, 1.67, 1.50, and then 1.33 for all subsequent dose-levels. In this form, the incremental ratio tends to a smaller constant number: 1.33.

Given that the so-called modified Fibonacci sequence remains the most popular dose increment determination scheme, but is also a non-standardized method, we carried out a literature review over a ten-year period of published dose-finding phase I trials investigating one drug to better characterize this series.

## Methods

The reports, considered for the present study, were extracted from a large database of 327 phase I trials (311 manuscripts) published between January 1997 and December 2008 in five major journals in the field of oncology(*Annals of OncologyClinical Cancer ResearchEuropean Journal of CancerInvestigational New Drugs* and *Journal of Clinical Oncology;* see Additional file 1). We have used Pubmed-Medline and the following keywords: “dose-seeking”, “phase 1 trial” and “cancer”. From these 327 trials, 197 were single-agent dose-seeking trials. From each report, we extracted the method used for guiding the dose-escalation and the result of the trial. “Failure of the trial” described a study failing to determine a phase-II-recommended-dose. The dose-increment guidance was classified into five categories: modified Fibonacci sequence, pharmacologically guided method [6], constant ratio incremental >+2.0 (aggressive increment, including most of accelerated titration designs [7]), constant ratio incremental <1.33 (prudent increment) and other methods (including bayesian approaches [8]). Among studies which used a modified Fibonacci sequence, we extracted the precise dose used at each dose-level. By convention, we have considered the actual dose by successive cohorts even if de-escalation occurred or the sponsor decided to explore an intermediate dose-level. De-escalation have been seen in only two trials. We then calculated for every dose-level: the mean dose administered at each dose level, its standard derivation, the median and the range. We then calculated the ratio of the median increments. For example, the ratio of median increment at dose-level 3 was the ratio of the median dose at level 3 over the median dose at level 2 across the studies which included at least these both dose-levels.

Lastly we plotted the mean incremental ratio (±95% confidence interval) on the log scale for the trial data compared to the incremental ratios of the genuine and modified Fibonacci series.

## Results

**Figure 1:**
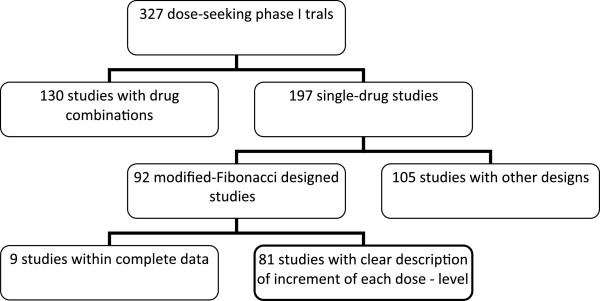
Pattern of 327 dose-seeking phase I trials.

Figure 1 describes the pattern of studies. The modified-Fibonacci-sequence was the most common method of dose-escalation (92/197, 46%). The contemporary studies still rarely used sophisticated designs such as Bayesian and pharmacologically guided dose escalation ( Table 1). The median number of dose-levels explored was 5 (range, 2–12). Four studies actually investigated only 2 dose-levels (Table 2).

**Table 1:** Dose-escalation-methods and results of 198 single-agent dose-seeking phase I trials

		**Guidance for the number of patients enrolled by dose-levels**	
		**Algorithm-based methods (such as 3 + 3) n (%)**	**Model-based methods (such as CRM) n (%)**
**Guidance for the dose-escalation**	Modified-Fibonacci sequence	92 (46.4)	0 (0)
	Fixed increment: <+33%	28 (14.1)	0 (0)
	Fixed increment: >100%	62 (1.3)	1 (0)
	PK-guided	3 (1.5)	1 (0)
	Other	7 (3.5)	4 (2)

**Table 2:** Dose and incremental ratios in 81 modified-Fibonacci-sequence designed studies

	**Planned dose in**		**Actual doses**			
**Dose-level (*n*of studies reaching the dose-level)**	**Genuine Fibonacci sequence**	**Modified Fibonacci sequence**	**Median of actual dose**	**Min. of actual dose**	**Max. of actual dose**	**Mean (± SD) of actual dose**
**1(81)**	1	1.0	1	-	-	-
**2(81)**	2	2.0	2.00	0.80	10	1.82 (0.65)
**3 (77)**	3	3.3	3.00	0.80	3.33	4.08 (1.65)
**4 (67)**	5	5.1	4.00	0.97	10.00	9.23 (3.12)
**5 (58)**	8	6.60	5.32	1.20	33.33	17.61 (4.71)
**6 (43)**	13	8.80	7.80	0.90	56.25	34.49 (6.89)
**7 (32)**	21	11.8	11.93	1.86	80.00	67.96 (9.77)
**8 (19)**	34	15.6	15.78	6.67	128.00	35.64 (5.43)
**9 (13)**	55	20.8	19.50	13.33	256.00	78.11 (8.69)
**10 (7)**	89	27.8	23.00	18.00	512.00	133.38 (12.69)
**11 (5)**	144	36.8	28.89	23.50	1024.00	227.16 (17.85)
**12 (3)**	233	49.0	38.44	35.83	2048.00	707.43 (29.89)
**13 (0)**	377	65.2	-	-	-	-

*n*: number of studied reaching the specified dose-level; SD: standard deviation.

The term modified-Fibonacci-sequence covered a vast range of actual doses (Table 2). For example, the actual increment at the fifth dose-level varied from 1.20 to 6.00. For every dose-level the observed dispersion of the actual increment was large (Table 2).

**Figure 2:**
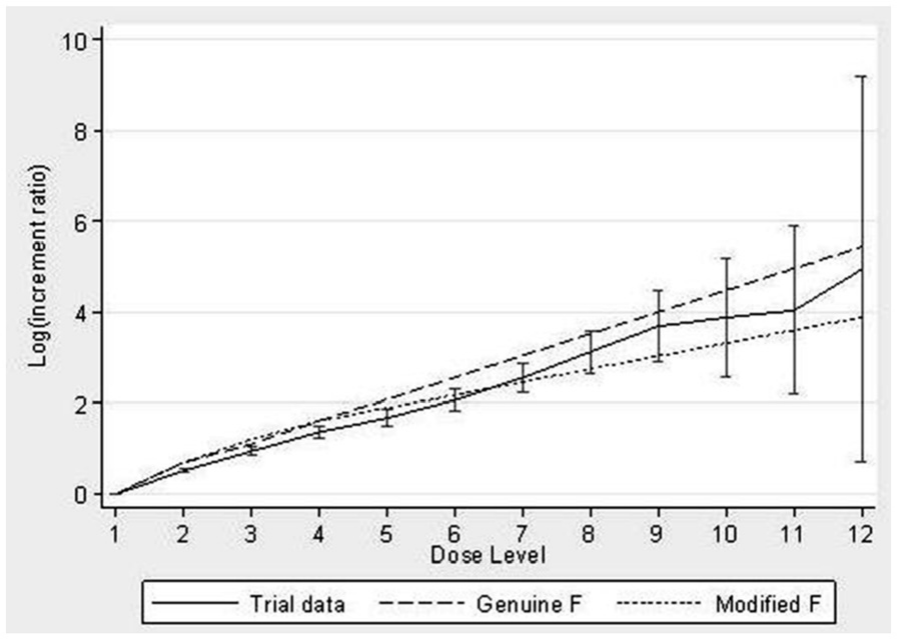
Comparison between actual sequence, genuine Fibonacci sequence and modified Fibonacci sequence.

The actual sequences used in the trials differed from the genuine Fibonacci sequence and the modified one by several points. The median actual dose was far from the genuine Fibonacci sequence, with values lower than expected starting from the fifth dose-level (5.32 instead of 8) and then huge differences in later dose-levels (38.44 instead of 233 at the 12th dose-level, see Figure 2 and Table 2). The median of actual increments was about 2.00, 1.50, 1.33, 1.33, 1.33, 1.33, 1.30, 1.35, 1.32, 1.24 and 1.25 (Table 3). This sequence did not tend to a constant number (see 4th column of Table 3).

**Table 3:** Incremental ratios

**Dose-levels**	**Incremental ratio in genuine Fibonacci sequence**	**Incremental ratio in Modified Fibonacci sequence**	**Incremental ratio of median dose in 81 phase I**
**1**	-	-	-
**2**	2.000	2.000	2.000
**3**	1.500	1.670	1.500
**4**	1.667	1.500	1.333
**5**	1.600	1.333	1.333
**6**	1.615	1.333	1.333
**7**	1.619	1.333	1.333
**8**	1.617	1.333	1.300
**9**	1.618	1.333	1.350
**10**	1.618	1.333	1.320
**11**	1.618	1.333	1.240
**12**	1.618	1.333	1.250
**13**	1.618	1.333	-

The mean incremental ratios followed the modified Fibonacci series more closely than the genuine series and they were statistically lower than the genuine series for dose levels 2 to 7 and significantly lower than the modified series for dose levels 2 to 5 (Figure 2). From dose levels 8 onwards, the mean incremental ratios were contained within the two series.

Moreover, in 13 studies (16%), at least one increment was significantly higher (e.g. >50%) than the increment planned with the modified-Fibonacci series.

## Discussion

Our analysis highlights the following facts. Most of contemporary dose-seeking phase I trials remain guided by the “modified Fibonacci sequence” (about 50%, Table 1). This term includes a vast variation in doses (from -20% to +208.3%, Table 2). Moreover, the actual series does not tend to a constant incremental ratio as expected from the modified Fibonacci sequence (Table 2) The dose-escalation is slower than planned by the genuine and modified Fibonacci sequence (Figure 2).

The algorithm-based designs tend to be more complex than the model-based-designs. Most of these new designs do not use the Fibonacci series. For example, Simon et al have proposed different designs called accelerated titration design, in which the dose doubling is used until there is one dose-limiting toxicity or two patients with at least two grade II toxicities at least possibly related to drug when a 40% increment is used [7]. The Bayesian designs do not use the Fibonacci series [8,9]. For example, the Bayesian logistic regression model with escalation with overdose control (EWOC) uses a model-based approach to determine the potential unsafe doses. The maximum dose increment may be capped at a doubling but a smaller dose increment can be used according to the specified overdose control or if there are other safety concerns. These approaches let the emerging data determine the future dose increment subject to a cap rather than a fixed algorithm [9]. Based on the observed toxicity profiles experienced in the trial, the continual reassessment method estimates the dose-level closest to the target (eg. 33% of patients presenting with dose limiting toxicities) and recommends treating the next patient at that dose level (without skipping dose levels). This implies an a priori specification of the drug toxicity profile characterized by a dose-response relationship for the drug and requires real-time data-collection, data-management and data-analysis of the toxicities used for the DLT definitions [8]. This process may slow down the completion of the trial. PK-guided designs require a validated, sensitive and specific drug assay. PK-guided dose-escalation also implies a relationship between plasma drug concentration and toxicity profile. This process also implies permanent sample collection, shipping, biological analysis and data-analysis. This may also slow down the trial [6]. As previously reported [1,2,10], about 50% of contemporary phase I trials still use the “Fibonacci series”; in our study, PK-guided dose-escalation or continual reassessement method are used in 3 and 2% of the trials, respectively. There are many reasons explaining that Fibonacci series remains largely used. First, this is one of the first described methods. This is easy to understand for the patient, the investigator and the regulatory bodies (such as Ethics Committee). The Fibonacci series is apparently cautious with a very progressive dose-escalation. Lastly, the implementation of this method does not need extensive statistical or biological work as compared to Bayesian approaches or PK-guided dose-escalation. However, this method is very rudimentary. Several analyses have demonstrated that Bayesian approaches, such as Continual Reassessment Method or EWOC reduce the number of patients treated at very low doses far from doses with potential therapeutic effect [2,9,10]. Some authors suggest than Fibonacci series is preferred when the dose-toxicity curve in animal toxicology is steep [10].

The main limitation of the present analysis is inherent to its retrospective nature. This includes a publication bias that implies a selection of manuscripts issued in journals with higher impact factors and consequently the trials with “positive” results.

## Conclusion

Modified Fibonacci series remains largely used [1,11]. The actual dose escalation referring to the “modified Fibonacci scheme” is a dose-escalation term close to and intermediate between the genuine and the “modified Fibonacci” schemes (Figure 2). In practice, however, the modified Fibonacci sequence, the most popular dose-escalation guidance scheme in dose-seeking phase I trials is a term which is too vague and should be avoided. The precise description of each dose-level in the study report should be certainly better than the use of this ambiguous term [5].

## Competing interests

The authors declare that they have no competing interests.

## Authors’ contributions

NP collected the data and participated to the statistical analysis. NP and AK conceived the study and supervised the statistical analysis. All authors read and approved the final manuscript.

## Authors’ information

NP is medical oncologist and head of the General Oncology Department at the Oscar Lambret Cancer Center. His main fields of expertise are treatment of rare tumors (including, adult sarcoma and cancer of unknown primary site) and early phase trials. AK is methologist and biostatistician. His main fields of expertise are development and validation of prognostic/predictive model and early phase trials methodology.

## Pre-publication history

The pre-publication history for this paper can be accessed here:

http://www.biomedcentral.com/1471-2288/12/103/prepub

## Supplementary Material

Additional file 1 List of the analyzed trials. Click here for file

## References

[B1] Rogatko A Schoeneck D Jonas W Tighiouart M Khuri FR Porter A Translation of innovative designs into phase I trials J Clin Oncol 2005 25 4982 6 17971597 10.1200/JCO.2007.12.1012

[B2] Ratain MJ Mick R Schilsky RL Siegler M Statistical and ethical issues in the design and conduct of phase I and II clinical trials of new anticancer agents J Natl Cancer Inst 1993 85 1637 43 10.1093/jnci/85.20.1637 8411243

[B3] Storer BE Crowley J, Hoering A, Ankerst D Choosing a phase I design Handbook of statistics in Clinical Oncology 2005 Second Edition Chapman & Hall/CRC Press, Taylor and Francis Group LLC, 600 Broken Parkway, NW, Suie 300, Boca Raton, FL 59 Third edition April 2012

[B4] Omura GA Modified Fibonacci search J Clin Oncol 2003 21 3177 79 10.1200/JCO.2003.99.058 12915613

[B5] Omura GA Phase 1 Dose-finding trials and Fibonacci Clin Cancer Research 2006 12 321 10.1158/1078-0432.CCR-05-1762 16397058

[B6] Collins JM Grieshaber CK Chabner BA Pharmacologically guided phase I clinical trials based upon preclinical drug development J Natl Cancer Inst 1990 82 1321 26 10.1093/jnci/82.16.1321 2143234

[B7] Simon R Freidlin B Rubinstein L Arbuck SG Collins J Christian MC Accelerated Titration Designs for phase I clinical trials in oncology J Natl Cancer Inst 1997 89 1138 47 10.1093/jnci/89.15.1138 9262252

[B8] O’Quigley J Sloan LR Continual reassessment method: A likelihood approach Biometrics 1996 52 673 84 10.2307/2532905 8672707

[B9] Babb J Rogatko A Zacks S Cancer Phase I clinical trials: efficient dose escalation with overdose control Stat Med 1998 17 1103 20 10.1002/(SICI)1097-0258(19980530)17:10<1103::AID-SIM793>3.0.CO;2-9 9618772

[B10] Zelen M Kufe DW, Pollock RE, Weischselbaum RR Theory and practive of clinical trials Holland-Frei Cancer Medicine 2003 Decker, Hamilton, Ontario BC

[B11] Dent SF Eisenhauer EA Phase I trial design: are new methodologies being put into practice? Ann Oncol 1996 7 561 66 10.1093/oxfordjournals.annonc.a010671 8879368

