# Supplementary material for: What does a modified-Fibonacci dose-escalation actually correspond to?
Source: BMC Med Res Methodol. 2012 Jul 23;12:103. doi: 10.1186/1471-2288-12-103 (PMC3538691; doi:10.1186/1471-2288-12-103)
Supplement: Additional file 1 — List of the analyzed trials. [file 1471-2288-12-103-S1.docx]

Supplementary material

List of the analyzed trials

1- Eder JP Jr, Garcia-Carbonero R, Clark JW, Supko JG, Puchalski TA, Ryan DP, et al. A phase I trial of daily oral 4’-N-benzoyl-staurosporine in combination with protracted continuous infusion 5-fluorouracil in patients with adavanced solid malignancies. Invest New Drugs 2004;22:139-50

2- Le LH, Erlichamn C, Pillon L, Thiessen JJ, Day A, Wainman N, et al. Phase I and pharmacokinetic study of fostriecin given as an intravenous bolus daily for five consecutive days. Invest New Drugs 2004;22:159-67

3- Gelmon K, Hirte H, Fischer B, Walsh W, Ptaszynski M, Hamilton M, et al. A phase 1 study of OSI-211 given as intravenous infusion days 1,2 and 3 every three weeks in patients with solid cancers. Invest New Drugs 2004;22:263-75

4- Hutson TE, Ganapathi R, Elson P, Mekhail T, Olencki T, Budd GT, et al. Phase I trial of vinorelbine and diphenylhydantoin in patients with refractory carcinoma. Invest New Drugs 2004;22:277-84

5- Curry EA III, Murry DJ, Yoder C, Fife K, Armstrong V, Nakshatri H, et al. Phase I dose escalation of feverfew with standardized doses of parthenolide in patients with cancer. Invest New Drugs 2004;22:299-305

6- Hoff PM, Saad ED, Pazdur R, Wolff R, Lassere Y, Bogaard KR, Abbruzzese JL. Phase I trial of combined irinotecan and oxaliplatin given every three weeks to patients with metastatic colorectal cancer. Invest New Drugs 2004;22:307-13

7- Majhail NS, Hussein M, Olencki TE, Budd GT, Wood L, Elson P, et al. Phase I trial of continuous infusion recombiant human interleukin-4 in patients with cancer. Invest New Drugs 2004;22:421-26

8- Chang SM, Kuhn J, Wen P, Greenberg H, Schiff D, Conrad C, et al. Phase I/pharmacokinetic study of CCI-779 in patients with recurrent malignant glioma on enzyme-inducing antiepileptic drugs. Invest New Drugs 2004;22:427-35

9- Schwartz GH, Jones CB, Garrison M, Patnaik A, Takimoto C, McGreery H, et al. A phase I and pharmacokinetic study of the nonpolyglutamable thymidylate synthetase inihibitor ZD9331 plus docetaxel in patients with adavanced solid malignancies. Invest New Drugs 2004;22:437-48

10- Undevia SD, Vogelzang NJ, Mauer AM, Janisch L, Mani S, Ratain MJ. Phase I clinical trial of CEP-2563 dihydrochloride, a receptor tyrosine kinase inhibitor, in patients with refractory solid tumors. Invest New Drugs 2004;22:449-58

11- Marshall JL, Kindler H, Deeken J, Bhargava P, Vogelzang NJ, Rizvi N, et al. Phase I trial of orally administered CEP-701, a novel neurotrophin receptor-linked tyrosine kinase inhibitor. Invest New Drugs 2005;23:31-7

12- Bjarnason GA, Charpentier D, Wong R, Goel R, Douglas L, Wlsh w, et al. Phase I study of tomudex and doxorubicin in patients with locally advanced inoperable or metastatic cancer (IND.98) Invest New Drugs 2005;23:51-6

13- El-Rayes BF, Ibrahim D, Shields AF, LoRusso PM, Zalupski MM, Philip PA. Phase I study of liposomal doxorubicin (Doxil) and cyclophosphamide in solid tumors. Invest New Drugs 2005;23:57-62

14- Goel R, Chouinard E, Stewart DJ, Huan s, Hirte H, Stafford S, et al. An NCIC CTG phaseI/pharmacokinetic study of matrix metalloproteinase and angiogenesis inhibitor BAY 12-9566 in combination with 5-fluorouracil/leucovorin. Invest New Drugs 2005;23:63-71

15- Murren J, Modiano M, Kummar S, Clairmont C, Egorin M, Chu E, et al. A phase I and pharmcokinetic study of VNP40101M, a new alkylating agent, in patients with advanced or metasttic cancer. Invest New Drugs 2005;23:123-35

16- Schmid P, Schweigert M, Beinert T, Flath B, Sezer O, Possinger K. Prolonged infusion of gemcitabine in advanced solid tumors: a phase-I-study. Invest New Drugs 2005;23:139-46

17- Goss G, Hirte H, Miller WH Jr, Lorimer IAJ, Stewart D, Batist G, et al. A phase I study of oral ZD 1839 given daily in patients with solid tumors: IND.122, a study of the Investigational New Drug Program of the National Cancer Institute of Canada Clinical Trials Group. Invest New Drugs 2005;23:147-55

18- Lim D, Morgan RJ, Akman S, Margolin K, Carr BI, Leong L, et al. Phase I trial of menadiol diphosphate (vitamin K3) in advanced malignancy. Invest New Drugs 2005;23:235-39

19- Wakelee H, Fischer GA. A phase I trial of irinotecan (CPT-11) with amifostine in patients with metastatic colorectal cancer. Invest New Drugs 2005;23:241-42

20- Chi KN, Chia SK, Dixon R, Newman MJ, Wacher VJ, Sikic B, et al. A phase I pharmacokinetic study of the P-glycoprotein inhibitor, ONT-093, in combination with paclitaxel in patients with advanced cancer. Invest New Drugs 2005;23:311-15

21- Schmid P, Flath B, Akrivakis K, Heilmann V, Mergenthaler HG, Sezer O, et al. Gemcitabine and mitoxantrone in metastatic breast cancer: a phase-I-study. Invest New Drugs 2005;23:349-56

22- Ottrrson GA, Lavelle J, Villalona-Calero MA, Shah M, Wei X, Chan KK, et al. A phase I clinical and pharmacokinetic study of fenretinide combined with pacliatxel and cisplatin for refractroy solid tumors. Invest New Drugs 2005;23:555-62

23- Argiris A, Kut V, Lung L, Avram MJ. Phase I and pharmacokinetic study of docetaxel, irinotecan, and celecoxib in patients with advanced non-small cell lung cancer. Invest New Drugs 2006;24:203-12

24- Wieder R, Novick SC, Hollis BW, Bryan M, Chanel SM, Owusu K, et al. Pharmacokinetics and safety of ILX23-7553, a non-calcemic-vitamin D3 analogue, in a phase I study of patients with advanced malignancies. Invest New Drugs 2003;21:445-52

25- Davis AJ, Gelmon KA, Siu LL, Moore MJ, Britten CD, Mistry N, et al. Phase I and pharmacologic study of the human DNA methyltransferase antisense oligoxynucleotide MG98 given as a 21-day continuous infusion every 4 weeks. Invest New Drugs 2003;2&:85-97

26- Dees EC, Rowinsky EK, Noe DA, O’Reilly S, Adjei AA, Elza-Brown K, et al. A phase I and pharmacologic study of pyrazoloacrine and cisplatin in patients with advanced cancer. Invest New Drugs 2003;21:75-84

27- Gadgeel SM, Boinpally RR, Heilbrun LK, Wozniak A, Jain V, Redman B, et al. A phase I clinical trial of spiramycin derivative (NSC 650426) using a phase I accelerated titration “2B” design. Invest New Drugs 2003;21:63-74

28- Garcia AA, Bookman MA, Rodriguez-Rodriguez L, Mutch DG, Look KY. Phase I escalation of gemcitabine combined with protracted oral etoposide in gynecologic malignancies. A Gynecologic Oncology Group Study. Invest New Drugs 2002;20:383-87

29- Morgan-Meadows S, Thomas JP, Mulkerin D, Berlin JD, Bailey h, Binger K, et al. Phase I study of eniluracil, oral 5-fluorouracil and gemcitabine in patients with advanced malignancy. Invest New Drugs 2002;20:377-82

30- Perez-Zincer F, Olencki T, Budd GT, Peereboom D, Elson P, Bukowski RM. A phase I trial of weekly gemcitabine and subcutaneous ineterferon alpha in patients with refractory renal cell carcinoma. Invest New Drugs 2002;20:305-10

31- Berlin JD, Alberti DB, Arzoomanian RZ, Feierabend CA, Simon KJ, Binger KA, et al. A phase I study of gemcitabine, 5-fluorouracil and leucovorin in patients with advanced, recurrent, and/or metastatic solid tumors. Invest New Drugs 1999;16:325-30

32- RaBmann I, Thodtmann R, Mross M, Huttman A, Berdel WE, Manegold Ch, et al. Phase I and pharmacokinetic trial of the podophyllotoxin derivative NK611 administered as intravenous short infusion. Invest New Drugs 1999;16:319-24

33- Masuada N, Negoro S, Takeda K, Takifuji N, Hirashima T, Yan T, et al. Phase I and pharmacologic study of oral (E)-2’-deoxy-2’-(fluoromethylene)cytidine: on a daily x 5-day schedule. Invest New Drugs 1999;16:245-54

34- Shade RJ, Pisters KMW, Huber MH, Fossella F, Perez-Soler R, Shin DM, et al. Phase I study of paclitaxel administered by ten-day continuous infusion. Invest New Drugs 1999;16:237-43

35- Cortes J, O’Brien S, Estey E, Giles F, Keating M, Kantarjan H. Phase I study of liposomal daunorubicin in patients with acute leucemia. Invest New Drugs 1999;17:81-7

36- Rischin D, Webster LK, Millward MJ, Toner GC, Nawaratne S, Ganju V, et al. A phase I and pharmacokinetic study of paclitaxel and eprubicin in advanced cancer. Invest New Drugs 1999;17:73-80

37- Tutsch KD, Arzoomanian RZ, Alberti D, Tombes MB, Feierbend C, Robins HI, et al. Phase I clinical and pharmacokinetic study of an one-hour infusion of ormaplatin (NSC 36812) Invest New Drugs 1999;17:63-72

38- Poplin E, Roberts J, Tombs M, Grant S, Rubin E. Leucovorin, 5-fluorouracil, and gemcitabine: a phase I study. Invest New Drugs 1999;17:57-62

39- Hirte H, Stewart D, Goel R, Chouinard E, Huan S, Stafford S, et al. An NCI-CTG phase I dose escalation pharmacokinetic study of the matrix metalloproteinase inhibitor BAY 12-9566 in combination with doxorubicin. Invest New Drugs 2005;23:437-43

40- Adjei AA, Reid JM, Erlichman C, Sloan JA, Pitot HC, Alberts SR, et al. A phase I and phamcologic study of pyrazoloacridine (NSC 366140) and carboplatin in patients with advanced cancer. Invest New Drugs 2002;20:297-304

41- Jones S, Hainsworth J, Burris HA III, Thompson D, Raefsky E, Johnson v, et al. Phase I study of JM-216 (an oral platinum analogue) in combination with paclitaxel in patients with advanced malignancies. Invest New Drugs 2002;20:55-61

42- LoRusso PM, Prakash S, Wozniak A, Flaherty L, Zalupski M, Shields A, et al. Phase I clinical trial of 5-fluoro-pyrimidone (5FP), an oral prodrug of 5-fluorouracil (5FU). Invest New Drugs 2002;20:63-71

43- Gelmon KA, Belanger K, Soulieres D, Britten c, Chia S, Charpentier D, et al. A phase I study of T900607 given once every 3 weeks in patients with advanced refractory cancer; National Cancer Institute of Canada Clinical Trials Group (NCIC-CTG) IND 130. Invest New Drugs 2005;23:445-53

44- Streiff RR, Bender JF. Phase I study of N1-N11-diethylnorspermine (DENSPM) administered TID for 6 days in patients with advanced malignancies. Invest New Drugs 2001;19:29-39

45- Blanke CD, Stipanov M, Morrow J, Rothenberg M, Chinery R, Shyr Y, et al. A phase I study of vitaminE, 5-fluorouracil and leucovorin for advanced malignancies. Invest New Drugs 2001;19:21-7

46-Giles F, Cortes J, Garcia-Manero G, Kornblau s, Estey e, Kwari M, et al. Phase I study of irofulven (MG 114), an acylfulvene illudin analog, in patients with cute leukaemia. Invest New Drugs 2001;19:13-20

47- Prakash S, Foster BJ, Meyer M, Wozniak A, Heilbrun LK, Flaherty L, et al. Chronic oral administration of CI-994: a phase I study. Invest New Drugs 2001;19:1-11

48- Luftner D, Flath B, Akrivakis C, Mergenthaler HG, Ohnmacht U, Arning M, et al. Gemcitabine plus dose-escalated epirubicin in advanced breast cancer: results of a phase I study. Invest New Drugs 1998;16:141-6

49- Davis SK, Dowlati A, O’Keefe S, Cline-Burkhardt M, Pelley RJ, Borden E, et al. Phase I trial of three-weekly docetaxel, carboplatin and oral lenalidomide (Revlimid) in patients with advanced solid tumors. Invest New Drugs 2007;25:211-16

50- Alousi AM, Boinpally R, Wiegand R, Parcment R, Gadgeel S, Heilbrun, et al. A phase 1 trial of XK469: toxicity profile of a selective topoisomerase IIβ inhibitor. Invest New Drugs 2007;25:147-54

51- Hageboutros A, Hudes GR, Greene F, LaCreta FP, Brennan J, O’Dwyeer PJ. Phase I trial of fluorouracil modulation by N-phosphonacetyl-L-aspartate and 6-methylmercapopurine ribonucleoside (MMPR), and leucovorin in patients with advanced cancer. Invest New Drugs 1997;15:139-45

52- Brand R, Capadano M, Tempero M. A phase I trial of weekly gelcitabine administered as a prolongad infusión in patients with pancreatic cancer and other solid tumors. Invest New Drugs 1997;15:331-41

53- Hainsworth JD, Utley SM, Greco FA. Phase I study of high dose etoposide phosphatase with filgrastim (G-CSF) in the treatment of advanced refractory malignancies. Invest New Drugs 1997;15:325-29

54- Schiller JH, Neuberg D, Burns D, Ritch P, Larson M, Lewitt M, et al. An Eastern Cooperative Oncology Group phase I trial of all-trans-retinoic acid and interferon-alpha: E2Y92. Invest New Drugs 1997;15:319-24

55- Delaunoit T, Burch PA, Reid JM, Camoriano JK, Kobayash T, Braich TA, et al. A phase I clinical and pharmacokinetic study of CS-682 administered orally in advanced solid tumors. Invest New Drugs 2006;24:327-33

56- Schwartzmann G, DiLeone LP, Horowitz M, Schunemann D, Cancella A, Pereira AS, et al. A phase I trial of the bombesin/gastrin-releasing peptide (BN/GRP) antagonist RC3095 in patients with advanced solid malignancies. Invest New Drugs 2006;24:403-12

57- El-Rayes BF, Gadgeek S, Parchment R, Lorusso P, Philip PA. A phase I study of flavopiridol and docetaxel. Invest New Drugs 2006;24:305-10

58- Hilgers W, Faivre S, Chieze S, Alexandre J, Lokiec F, Goldwasser F, et al. A phase I and pharmacokinetic study of irofulven and cisplatin administered in a 30-min infusion every two weeks to patients with advanced solid tumors. Invest New Drugs 2006;24:311-19

59- Olencki T, Malhi S, Mekhail T, Dreicer R, Elson P, Wood L, et al. Phase I trial of thalidomide and interleukine-2 in patients with metastatic reanal cell carcinoma. Invest New Drugs 2006;24:321-26

60- Cho CD, Fisher GA, Halsey J, Sikic B. Phase I study of gefitinib, oxaliplatin, 5-fluorouracil, and leucovorin (IFOX) in patients with advanced solid malignancies. Invest New Drugs 2006;24:117-23

61- Goel S, Desai K, Macpinlac M, Wadler S, Goldberg G, Fields A, et al. A phase I safety and dose escalation trial of docetaxel combined with GEM 231, a second generation antisense oligonucleotide targeting protein kinase A R1α in patients with advanced solid cancers. Invest New Drugs 2006;24:125-34

62- Ghesquières H, Faivre S, Djafari L, Pautier P, Lhommé C, Lozahic S, et al. Phase I dose escalation study of pegylated doxorubicin (Caelyx) in combination with topotecan in patients with advanced malignancies. Invest New Drugs 2006;24:413-21

63- Mekhail T, Kaur H, Ganapathi R, Budd GT, Elson P, Bukowski RM. Phase 1 trial of Anvirzel in patients with refractory solid tumors. Invest New Drugs 2006;24:423-27

64- Gilbert J, Carducci MA, Baker SD, Dees EC, Donehower R. A phase I study of the oral antimetabolite, CS-682, administered once daily 5 days per week in patients with refractory solid tumor malignancies. Invest New Drugs 2006;24:499-508

65 and 66- James J, Murry DJ, Treston AM, Storniolo AM, Sledge GW, Sidor C, et al. Phase I safety, pharmacokinetic and pharmacodynamic studies of 2-methoxyestradiol alone or in combination with docetaxel in patients with locally recurrent or metastatic breast cancer. Invest New Drugs 200-;25:41-8

67- Camacho LH, Olson J, Tong WP, Young CW, Spriggs DR, Malkin MG. Phase I dose escalation clinical trial of phenylbutyrate sodium administered twice daly to patients with advanced solid tumors. Invest New Drugs 2007;25:131-8

68- Skarin NT, Lathia CD, Benson L, Grove WR, Thomas S, Roca J, et al. A phase I trial and pharmacokinetic evaluation of CI-980 with advanced solid tumors. Invest New Drugs 1997;15:235-46

69- Flaig TW, Gustafson DL, Su LJ, Zirolli JA, Crighton F, Harrison GS, et al. A phase I nd pharmacokinetic study of silybin-phytosome in prostate cancer patients. Invest New Drugs 2007;25:139-46

70- Creavan PJ, Perez R, Pendyala L, Meropol NJ, Loewen G, Levine E, et al. Unusual central nervous system toxicity in a phase I study of N1N11diethylnospermine in patients with advanced malignancy. Invest New Drugs 1997;15:227-34

71- Baetz T, Eisenhauer E, Siu L, MacLean M, Doppler K, Walsk W, et al. A phase I study of oral LY293111 given daily in combination with irinotecan in patients with solid tumours. Invest New Drugs 2007;25:217-225

72- Pazdur R, Lassere Y, Diaz-Canton E, Bready B, Ho DH. Phase I trial of Uracil-Tegafur (UFT) plus oral leucovorin: 14-day Schedule. Invest New Drugs 1997;15:123-28

73- Rowinsy EK, Flood WA, Sartorius SE, Bowling KM, Ettinger DS, et al. Phase I study of paclitaxel on a 3-hour schedule followed by carbooplatin in untreated patients with stage IV non-small cell lung cancer. Invest New Drugs 1997;15:129-38

74- Kuppens LELM, Witteveen PO, Schot M, Schuessler VM, Daehling A, Beijnen JH, et al. Phase I dose-finding and pharmacokinetic trial of orally administered indibulin (D-24851) to patients with solid tumors. Invest New Drugs 2007;25:227-35

75- Pollera CF, Ceribelli A, Crecco M, Oliva C, Calabresi F, et al. Prolongad infusión gemcitabine: a clinical phase I study at low- (300 mg/m²) and high-dose (875 mg/m²) levels. Invest New Drugs 1997;15:115-21

76- Goel S, Desai K, Karri S, Gollamudi R, Chaudhary I, Bulgaru A, et al. Pharmacokinetic and safety studu of weekly irinotecan and oral capecitabine in patients with solid cancers. Invest New Drugs 2007;25:37-45

77- Chen EX, Batist G, Siu LL, Bangash N, Maclean M, McIntosh L, et al. Phase and pharmacokinetic study of Bay 38-3441, a camptothecin glyconjugate, administered as a 30-minute infusion daily for five f=days every 3 weeks in patients with advanced solid malignancies. Invest New Drugs 2005;23:455-65

78- Edelman MJ, Gandara DR, Perez EA, Lau D, Lauder I, Turrell C, et al. Phase I trail of edatrexate plus carboplatin in advanced solid tumots : amelioration of dose-limiting mucositis by ice chip cryotherapy. Invest New Drugs 1998;16:69-75

79- Burris HA III, Raymond E, Awada A, Kuhn JG, O’Rourke TJ, Brentzel J, et al. Pharmacokinetic and phase I studies of brequinar (DUP 785;NSC 368390) in combination with cisplatin in patients with advanced malignancies. Invest New Drugs 1998;16:19-27

80- Rodriguez GI, Kuhn JG, Weiss G, De La Cruz P, New P, Fields SM, et al. A phase I and pharmacokinetic trial of terephthalamidine (NSC 57155) as a 120-hour continuous infusion. Invest New Drugs 1998;16:57-67

81- Advani R, Lum BL, Fischer GA, Halsey J, Geary RS, Holmlund JT, et al. A phase I trial of aprinocarsen (ISI 3521/LY900003), an antisnse inhibitor of protein kinase C-alpha administered as a 24-hour weekly infusion schedule in patients with advanced cancer. Invest New Drugs 2005;23:467-77

82- Hayes TG, Falchook GF, Varadhachary GR, Smith DP, Davis LD, Dhingra HM, et al. Phase I trial of oral talactoferrin alfa in refactory solid tumors. Invest New Drugs 2006;24:233-40

83- Maroun JA, Stewaet D, Verma S, Eisenhauer E. Phase I clinical study of didemnin B. Invest New Drugs 1998;16:51-6

84- Johnson P, Geldart T, Fumoleau P, Pinel MC, Nguyen L, Judson I. Phase I study of vinflunine administered as a 10-minute infusion on days 1 and 8 every 3 weeks. Invest New Drugs 2006;24:223-31

85- Thompson J, Pratt CB, Stewart CF, Avery L, Bowman L, et al. Phase I study of BMP 840 in pediatric patients with refractory solid tumors. Invest New Drugs 1998;16:45-9

86- Salzberg M, Pless M, Rochlitz c, Ambrus K, Scigalla P, Hermann R. A phase I study of oral SU5416 in patirnts with advanced solid tumors: a drug inducing its clearance. Invest New Drugs 2006;24:299-304

87- Peacock NW, Burris HA III, Dieras V, Smith L, Rodriguez GL, Eckardt JR, et al. A phase I trial of vinorelbine in combination with mitoxantrone in patients with refractory solid tumors. Invest New Drugs 1998;16:37-43

88- Garcia AA, Iqbal S, Quinn D, Edwards S, Lenz HJ, Weber J. Phase I clinical trial of weekly docetaxel and exisulind, a novel inducer of apoptosis. Invest New Drugs 2006;24:79-83

89- Einzing AI, Wiernik PH, Wadler S, Kapan j, Benson LT, Tentoramano L, et al. Phase I study of paclitaxel (taxol) and granulocyte colony stimulating factor (G-CSF) in patients with unresectable malignancy. Invest New Drugs 1998;16:29-36

90- MacKenzie MJ, Hirte HW, Siu LL, Gelmon K, Ptaszynski M, Fischer B, Eisenhauer E. A phase I study of OSI-211 and cisplatin as intravenus infusions given on days 1, 2 and 3 every 3 weeks in patients with solidcancers. Ann Oncol 2004;15:665-70.

91- Marshall J, Chen H, Yang D, Figueira M, Bouker KB, Ling Y, Lippman M, Frankel SR, Hayes DF. A phase I trial of a Bcl-2 antisense (G3129) and weekly docetaxel in patients with advanced breast cancer and other solid tumors. Ann Oncol 2004;15:1274-83.

92- Awada A, Giacchetti S, Gerard B, Eftekhary P, Lucas C, de Valeriola D, et al. Clinical phase I and pharmacokinetic study of S 16020, a new olivacine derivative: report of three infusion schedules. Ann Oncol 2002;13:1925-34

93- Bennouna J, Fumoleau P, Armand JP, Raymond E, Campone M, Delgado FM, et al. Phase I and pharmacokinetic study of a new vinca alkaloid vinflunine administered as a 10-min infusión every 3 weeks in patients with advanced solid tumours. Ann Oncol 2003;14:630-7.

95- Mitchell PLR, Basser R, Chipman M, Grigg A, Cebon J, Davis ID, et al. A phase I dose-escalation study of docetaxel with granulocyte colony-stimulating factor support in patients with solid tumours. Ann Oncol 2003; 14:788-94.

96- Punt CJA, Boni J, Bruntsch U, Peters M, Thielbert C. Phase I and pharmacokinetic study of CCI-779, a novel cytostatic cell-cycle inhibitor, in combination with 5-fluorouracil and leucovorin in patients with advanced solid tumors. Ann Oncol 2003;14:931-37.

97- Schrijvers D, Bos AME, Dyck J, de Vries EGE, Wanders J, Roelvink M, et al. Phase I study of MEN-10755, a new anthracycline in patients with solid tumourd: a report from the European Organization for Research and Treatment of Cancer, Early Clinical Studies Group. Ann Oncol 2002;13:385-91.

98- Simpson AB, Calvert PM, Sludden JA, Boddy AV, Griffin MJ, Shatzlein A, et al. Topotecan in combination with carboplatin: a phase I trial evaluation of two treatment schedules. Ann Oncol 2002;13:399-402.

99- Gemcitabine and oxaliplatin for patients with advanced or metastatic pancreatic cancer: a North Central Treatment Group (NCCTG) phase I study. Ann Oncol 20002;13:553-57

100- Mayer F, Hartmann JT, von Pawel J, Beck J, Schroeber M, Boehlke I, et al. A phase I study of oral uracil-ftorafur plus folinic acid in combination with weekly paclitaxel in patients with solid tumors. Ann Oncol 2002;13:755-59.

101- Sessa C, Cuvier C, Caldiera S, Bauer J, Van den Bosch S, Monnerat C, et al. Phase I clinical and pharmacokinetic studies of the staxoid derivative RPR 109881A administreted as a 1-hour or 3-hour infusion in patients with advanced solid tumors. Ann Oncol 2002;13:1140-50.

102- Evans TRJ, Pentheroudakis G, Paul J, McInnes A, Blackie R, Raby N, et al. A phase I and pharmacokinetic study of capecitabine in combination with epirubicin and cisplatin in patients with inoperable oesophago-gastric adenocarcinoma. Ann Oncol 2002;13:1469-78

103- Golberg RM, Kaufmann SH, Atherton P, Sloan JA, Adjei AA, Pitot HC, et al. A phase I study of sequential irinotecan and 5-fluorouracil/leucovorin. Ann Oncol 2002;13:1674-80.

104- Wadler S, Makower D, Clairmont C, Lambert P, Fehn K, Sznol M. Phase I and pharmacokinetic study of rinonucleotide reductase inhibitoer, 3-aminopyridine-2-carboxaldehyde thiosemicarbazone, administered by 96-hour continuous infusion. J Cln Oncol 2004 ;22 :1553-63.

105- Raymond E, Alexandre J, Faivre S, Vera K, Materman E, Boni j, et al. Safety and pharmacokinetics of escalated doses of weekly intravenous infusion of CCI-779, a novel mTOR inhibitor, in patients with cancer. J Clin Oncol 2004;22:2336-47.

106- Papandreou CN, Daliani DD, Nix D, Yang H, Madden T, Wang X, et al. Phase I trial of a proteasome inhibitor bortezomib in patients with advanced solid tumors with observations in androgen-independent prostate cancer. J Clin Oncol 2004;22:2108-21

107- Hoekstra R, de Vos FYFL, Eskens FALM, de Vries EGE, Uges DRA, Knight R, et al. Phase I study of the thrombospondin-1-mimetic angiogenesis inbitor ABT-510 with 5-fluorouracil and leucovorin : a safe combination. Eur J Cancer 2006;42:467-72.

108-Mross K, Drves J, Mûller M, Medinger M, Marmé D, Hennig J, et al. Phase I clinical and pharmacokinetic study of PTK/ZK a multiple VEGF receptor inhibitor, in patients with liver metastases from solid tumours. Eur J Cancer 2005;41:1291-99

109- Campone M, Fumoleau M, Delecroix V, Deporte-Fety R, Perrocheau G, Vernillet L, et al. Phase I dose-finding and pharmacokinetic study of docetaxel and vinorelbine as first-line chemotherapy for metastatic breast cancer. Ann Oncol 2001;12:909-18.

110- Borchmann P, Schnell R, Knippertz R, Staak JO, Camboni GM, Bernareggi A, et al. Phase I study of BBR 2778, a new aza-anthracenedione, in advanced or refractory non-Hodgkin’s lymphoma. Ann oncol 2001;12:661-67.

111- Nyman DW, Campbell KJ, Hersch E, Long K, Richardson K, Trieu V, et al. Phase I and pharmacokinetics trial of ABI-007, a novel nonoparticle formulation of paclitaxel in patients with advanced nonhematologic malignancies. J Clin Oncol 2005;23:7785-93.

112- Kreis W, Budman DR, Fetten J, Gonzales AL, Barille B, Vinciguerra V. Phase I trial of the combination of daily estramustine phosphate and intermittent docetaxel in patients with metastatic hormone refractory carcinoma. Ann Oncol 1999;10:33-38

113- Schoffski P, Seeland G, Engel H, Grunwald V, Paul H, Merkle K, et al. Weekly administration of bendamustine: a phase I study in patients with advanced progressive solid tumours. Ann Oncol 2000;11:729-34

114- Sausville EA, Arbuck SG, Messmann R, Headlee D, Bauer KS, Lush RM, et al. Phase I trial of 72-hour continuous infusion UCN-01 in patients with refractory neoplasms. J Clin Oncol 2001;19:2319-33

115- Dowlati A, Hoppel CL, Ingalls ST, Majka S, Li X, Sedransk N, et al. Phase I clinical and pharmacokinetic study of rebeccamycin anlog NSC 655649 given daily for five consecutive days. J Clin Oncol 2001;19:2309-18.

116- Hidalgo M, Aylesworth C, Hammond LA, Britten CD, Weiss G, Stephenson J, et al. Phase I and pharmacokinetic study of BMS-184476, a taxane with greater potency and solubility than paclitaxel. J Clin Oncol 2001;19:2493-503.

117- Shilsky R, Bertucci D, Vogelzang NJ, Kindler HL, Ratain MJ. Dose-escalating study of capecitabine plus gemcitabine combination therapy in patients with advanced cancer. J Clin Oncol 2002;20:582-87.

118- Pignatta S, Varriale E, Casella G, Iodice F, De Placido G, Perrone F, et al. A phase I study of gemcitabine and epirubicin for the treatment of platinum-resistant or refractory advanced ovarian cancer. Ann Oncol 2000;11:613-16.

119- Caponigro F, Comella P, Budillon A, Bryce J, Avallone A, De Rosa V, et al. Phase I study of Caelyx (doxorubicin HCL, pegylated liposomal) in recurrent or metastatic head and neck cancer. Ann Oncol 2000;11:339-42.

120- Tolcher AW, Aylesworth C, Rizzo J, Izbicka E, Campbell E, Kuhn J, et al. A phase I of rhizoxin (NSC 332598) by 72-hour continuous intravenous infusion in patients with advanced solid tumors. Ann Oncol 2000;11:333-38.

121- Burstein HJ, Ramirez MJ, Petros WP, Clarke KD, Warmuth MA, Marcom PK, et al. Phase I study of doxil and vinorelbine in metastatic breast cancer. Ann Oncol 1999;10:1113-16

122- Dunphy FR, Dunleavy TL, Harrisson BR, Cantrell CL, Visconti JL, Pincus SM, et al. Phase I dose escalation study of topotecan combined with alternating schedules of paclitaxel and carboplatin in advanced solid tumors. Ann Oncol 2001;12:549-55.

123- ten Bokkel Huinink D, Tesselaar MET, Baatenburg de Jong RJ, Verschuur HP, Keizer HJ. A phase I study of fixed dose of cisplatin with increasing doses of raltitrexed (Tomudex) in the treatment of patients with locally advanced or metastatic squamous cell carcinoma of head and neck. Ann Oncol 2001;12:357-63

124- Rheingold SR, Hogarty D, Blaney SM, Zwiebel JA, Sauk-Schubert C, Chandula R, et al. Phase I trial of G3139, a bcl-2 antisense oligonucleotide, combined with doxorubicin and cyclophosphamide in children with relapsed solid tumors: a children’s Oncology Group Study. J Clin Oncol 2007;12:1512-18

125- Dragovich T, Gordon M, Mendelson D, Wong L, Modiano M, Sherry Chow HH, et al. Phase I trial of imexon in patients with advanced malignancy. J Clin Oncol 2007;13:1779-84

126- Aghajanian C, Burris HA III, Jones S, Spriggs DR, Cohen MB, Peck R, et al. Phase I study of the novel epothilone analog ixabepilone (BMS-247550) in patients with advanced solid tumors and lymphomas. J Clin Oncol 2007;25:1082-88

127- Whitlock JA, Krailo M, Reid JM, Ruben SL, Ames MM, Owen W, et al. Phase I clinical and pharmacokinetic study of flavopiridol in children with refractory solid tumors : a Children’s Oncology Group Study. J Clin Oncol 2005;23:9179-86.

128- Tolcher AW, Mita M, Meropol NJ, Von Mehren M, Patnaik A, Padavic K, et al. Phase I pharmacokinetic and biologic correlative stud of mapatumab, a fully human monoclonal antibody with agonist activity to tumor-necrosis factor-related apoptosis-inducing ligand receptor-1. J Clin Oncol 2007;25:1390-95.

129- Giles F, Verstovsek S, Thomas D, Gerson s, Cortes J, Faderl S, et al. Phase I study of cloretazine (VNP40101M), a novel sulfonylhydrazine alkylating agent, combined with cytarabine in patients with refractory leukaemia. Clin Cancer Res 2005;21:7817-24

130- Royce M, Hoff PM, Dumas P, Lassere Y, Lee JJ, Coyle J, et al. Phase I and pharmacokinetic of exatecan mesylate (DX-8951f): a novel campothecin analog. J Clin Oncol 2001;19:1493-500

131- Rivera E, Valero V, Syrewicz L, Rahman Z, Esteva FL, Theriault RL, et al. Phase I study of stealth liposomal doxorubicin in combination with gemcitabine in the treatment of patients with metastatic breast cancer. J Clin Oncol 2001;19:1716-22

132- Munster P, Buzdar A, Dhingra K, Enas N, Ni L, Major M, et al. Phase I study of a third-generation selective estrogene receptor modulator, LY353381.HC1, in metastatic breast cancer. J Clin Oncol 2001;19:2002-09

133- Pisters KMW, Newman RA, Coldman B, Shin DM, Khuri FR, Hong WK, et al. Phase I trial of oral green tea extract in adult patients with solid tumors. J Clin Oncol 2001;19:1830-38.

134- Ewesuedo RN, Lyer L, Das S, Koenig a, Mani S, Vogelzang NJ, et al. Phase I clinical and pharmacogenetic study of weekly TAS-103 in patients with advanced cancer. J Clin Oncol 2001;19:2084-90.

135- Hammond LA, Eckardt JR, Baker SD, Eckardt SG, Dugan M, Forral K, et al. Phase I and pharmacokinetic study of temozolomide on a daily-for-5-days schedule in patients with advanced solid malignancies. J Clin Oncol 1999;17:2604-13.

136- Villalona-Calero MA, Weiss GR, Burris HA, Kraynak M, Rodrigues G, Drengler RL, et al. Phase I and pharmacokinetic study of the oral fluoropyrimidine capecitabine in combination with paclitaxel in patients with advanced solid malignacies. J Clin Oncol 1999;17:1915-25

137- Herben VMM, Van Gijn R, Schelens JHM, Schot M, Lieverst J, Hillebrand MJX, et al. Phase I and pharmacokinetic study of a daily times 5 short intravenous infusion schedule of 9-aminocamptothecin in a colloidal dispersion formulation in patients with advanced solid tumors. J Clin Oncol 1999;17:1906-14

138- Herben VMM, Schellens JHM, Swart M, Gruia G, Vernillet L, Beijnen JH, et al. Phase I and pharmacokinetic study of irinotecan administered as a low-dose, continuous intravenous infusion over 14 days in patients with malignant solid tumors. J Clin Oncol 1999;17:1897-1905

139- Eckhardt SG, Rizzo J, Sweeney KR, Cropp G, Baker GC, Kraynak MA, et al. Phase I and pharmacologic study of the tyrosine kinase inhibitor SU101 in patients with advanced solid tumors. J Clin Oncol 1999;17:1095-1104.

140- Nemunaitis J, Holmlund JT, Kraynak M, Richards D, Bruce J, Ognoskie N, et al. Phase I evaluation of ISIS 3521, an Antisense oligodeoxynucleotide to protein kinase C-alpha, in patients with advanced cancer. J Clin Oncol 1999;17:3586-95.

141- Thodtmann R, Depenbrock H, Dumez H, Blatter J, Johnson RD, van Oosterom A, et al. Clinical and pharmacokinetic phase I study of multitargeted antifolate (LY231514) in combination with cisplatin. J Clin Oncol 1999;17:3009-16

142- Rizzieri DA, Bass AJ, Rosner GL, Gockerman JP, DeCastro CM, Petros WP, et al. Phase I evaluation of prolongad-infusion gemcitabine with mitoxantrone for relapsed or refractory acute leukemia. J Clin Oncol 2002;20:674-79.

143- Ebbinghaus S, Rubin E, Hersh E, Cranmer LD, Bonate PL, Fram RJ, et al. A phase I study of the dolastatin-15 analogue tasidotin (ILX651) administered intravenously daily for5 consecutive days every 3 weeks in patients with advanced solid tumors. Clin Cancer Res 2005;21:7807-16

144- Eskens FALM, Awada A, Cutler DL, de Jonge MJA, Luyten GPM, Faber MN, et al. Phase I and pharmacokinetic study of the oral farnesyl transferase inhibitor SCH 66336 given twice daily to patients with advanced solid tumors. J Clin Oncol 2001;19:1167-75

145- Schilder RJ, Gallo JM, Millenson MM, Bookman MA, Weiner LM, Rogatko A, et al. Phase I trial of multiple cycles of high-dose carboplatin, paclitaxel, and topotecan with peripheral-blood stem-cell support as front-line therapy. J Clin Oncol 2001;19:1183-94

146- Fracasso PM, Westerveldt P, Fears CA, Rosen M, Zuhowski EG, Cazenave LA, et al. Phase I study of paclitaxel in combination with a multidrug resistance modulator, PSC 833 (Valspodar), in refractory malignancies. J Clin Oncol 2000;18:1124-34.

147- Adjei AA, Erlichman C, Sloan JA, Reid JM, Reid JM, Pitot HC, et al. Phase I and pharmacologic study of sequences of gemcitabine an the multitargeted antifolate agent in patients with advanced solid tumor. J Clin Oncol 2000;18:1748-57

148- Malingré MM, Meerum Terwogt JM, Beijen JH, Rosing H, Koopman FJ, van Tellingen O, et al. Phase I and pharmacokinetic study of oral paclitaxel. J Clin Oncol 2000;18:2468-75

149- Ranson M, Hammond LA, Ferry D, Kris M, Tullo A, Murray PI, et al. ZD1839, a selective oral epidermal growth factor recptor-tyrosine kinase inhibitor, is well tolerated and active in patients with solid, malignant tumors : results of a phase I trial. J Clin Oncol 2002;20:2240-50.

151- Cloughesy TF, Kuhn J, Robins HI, Abrey L, Wen P, Fink K, Liberman FS, et al. Phase I trial of tipifarnib in patients with recureent malignant glioma taking enzyme-inducing antiepileptic drugs : a north americn brain tumor consortium study. J Clin Oncol 2005;23:6647-56.

152- Faivre S, Chièze S, Delbado C, Ady-Vago N, Guzman C, Lopez-Lazaro L, et al. Phase I and pharmacokinetic study of aplidine, a new marine cyclodepsipeptide in patients with adavanced malignancies. J Clin Oncol 2005;23:7871-80

153- Wirth LJ, Haddad RI, Linderman NI, Zhao X, Lee JC, Joshi VA, et al. Phase I study of gefitinib plus celecoxib in recurrent or metastatic squamous cell carcinoma og head and neck. J Clin Oncol 2005;23:6976-81

154- Ajani JA, Faust J, Ikeda K, Yao JC, Anbe H, Carr KL, et al. Phase I pharmacokinetic study of S-1 plus cisplatin in patients with advanced gastric carcinoma. J Clin Oncol 2005;23:6957-65

155- Fouadi M, Furhman WL, Chin T, Freeman BB III, Dudkin L, Stewart CF, et al. Phase I study of depsipeptide in padiatric patients with refractory solid tumors: a Children’s Oncology Group Report. J Clin Oncol 2006;24:3678-85

156- Knox JJ, Siu LL, Chen E, Dimitroulakos J, Kamel-Reid S, Moore MJ, et al. A phase I trial of prolonged administration of lovastatin in patients with recurrent or metastatic squamous cell carcinoma of the head and neck or the cervix. Eur J Cancer 2005;41:523-30.

157- Dittrich C, Petruzelka L, Vodvarka P, Gneist M, Janku F, Kysela T, et al. A phase I study of pemetrexed (ALIMTA) and cyclophosphamide in patients with locally advanced or metastatic breast cancer. Clin Cancer Res 2006;23:7071-78.

158- Patnaik A, Wood D, Tolcher AW, Hamilton M, Kreisberg JI, Hammond LA, et al. Phase I, pharmacokinetic, and biological study of erlotinib in combination with paclitaxel and carboplagtin in patients with advanced solid tumors. Clin Cancer Res 2006;7406-13

159- Mross K, Steinbild S, Baas F, Gmehling D, Radtke M, Voliotis D, et al. Results from an in vitro and a clinical/pharmacological phase I study with the combination irinotecan and sorafenib. Eur J Cancer 2007;43:55-63

160- Messersmith WA, Rudek MA, Baker SD, Zhao M, Collins C, Colevas AD, et al. Phase I study of continuous weekly dosing of dimethylaminobezoylphenylurea (BPU) in patients with solid tumours. Eur J Cancer 2007;43:78-86.

161- Baruchel S, Diezi M, Hargave D, Stempack D, Gammon J, Moghrabi A, et al. Safety and pharmacokinetics of temozolomide using a dose-escalation, metronomic schedule in recurrent paediatric brain tumours. Eur J Cancer 2006;42:2335-42

162- Sharma RA, Steward WPP, Daines CA, Knight RD, O’Byrne KJ, Dalgleish AG. Toxicity profile of the immunomodulatory analogue, lenalidomide: phase I clinical trial of three dosing schedules in patients with sold malignancies. Eur J Cancer 2006;42:2318-25

163- Izquierdo MA, Garcia M, Ponton JL, Martinez M, Valenti V, Navarro M, et al. A phase I clinical and pharmacokinetic study of paclitaxel and docetaxel given in combination in patients with solid tumours. Eur J Cancer 2006;42:1789-96

164- Sessa C, Vigano L, Grasselli G, Trigo J, Marimon I, Llado A, et al. Phase I clinical and pharmacological evaluation of the multi-tyrosine kinase inhibitor SU006668 by chronic oral dosing. Eur J Cancer 2006;42:171-8

165- Villalona-Calero MA, Blum JL, Jones SE, Diab S, Elledge R, Khoury P, et al. A phase I and pharmacologic study of capecitabine and paclitaxel in breast cancer patients. Ann Oncol 2001;12:605-14

166- Alberts SR, Erlichman C, Sloan J, Okuno SH, Burch PA, Rubin J, et al. Phase I trial of gemcitabine and CPT-11 given weekly for four weeks every six weeks. Ann Oncol 2001;12:627-31

167- Santini D, Virzi V, Vincenzi B, Rocci L, Leoni V, Tonini G. A phase I trial of fixed dose rate gemcitabine plus capeticabine in metastatic cancer patients. Ann Oncol 2007;18:576-80

168- Daw NC, Furman WL, Stewart CF, Iacono LC, Krailo M, Bernstein ML, et al. Phase I and pharmacokinetic study of gefitinib in children with refractrory solid tumors: a Children’s Oncology Group Study. J Clin Oncol 2005;23:6172-80.

169- Schoffski P, Breidenbach I, Krauter J, Bolte O, Stadler M, Ganser A, et al. Weekly 24 h infusion of aviscumine (rViscumin): A phase I study in patients with solid tumours. Eur J Cancer 2005;41:1431-38

170- Panasci L, Stinson SF, Melnychuk D, Sandor V, Miller WH Jr, Batist G, et al. SarCNU, a nitrosourea analog on a day 1, 5 and 9 oral schedule: a phase I and pharmacokinetic study in patients with advanced solid tumors. J Clin Oncol 2003;21:232-40.

171- Twelves C, Trigo JM, Jones R, De Rosa F, Rakhit A, Fettner S, et al. Erlotinib in combination with capecitabine and docetaxel in patients with metastatic breast cancer: a dose-escalation study. Eur J Cancer 2008;44:419-26

172- Awada A, Cardoso F, Fontaine C, Dirix L, De Grève J, Sotiriou C, Steinseifer J, et al. The oral mTO inhibitor RAD001 (everolimus) in combination with letrozole in patients with advanced breast cancer: results of a phase I study with pharmacokinetics. Eur J Cancer 2008;44:84-91

173- Rubin EH, Rothermel J, Tesfaye F, Chen T, Hubert M, Ho YY, et al. Phase I dose-finding study of weekly single-agent patupilone in patients with advanced solid tumors. J Clin Oncol 2005;23:9120-29

174- Thomas JP, Arzoomanian RZ, Alberti D, Marnocha R, Lee F, Friedl A, et al. Phase I Pharmacokinetic and pharmacodynamic study of recombinant human endostatin in patients with advanced solid tumors. J Clin Oncol 2003;21:223-231.

175- Fleming GF, Meropol NJ, Rosner GL, Hollis DR, Carson WE III, Caligiuri M, et al. A phase I trial of escalating doses of trastuzumab combined with daily subcutaneous interleukin 2: report of Cancer and eukemia Group B 9661. Clin Cancer Res 2002;8:3718-27

176- Adjei AA, Dy GK, Erlichman C, Reid JM, Sloan JA, Pitot HC, et al. A phase I trial of ISIS 2503, an antisense inhibitor of H-ras, in combination with gemcitabine in patients with advanced cancer. Clin Cancer Res 2003;9:115-23

177- Syed S, Takimoto C, Hidalgo M, Rizzo J, Kuhn JG, Hammond LA, et al. A phase I and pharmacokinetic study of Col-3 (Metastat), an oral tetracycline derivative with potent matrix metalloproteinase and antitumor properties. Clin Cancer Res 2004;10:6512-21

178- Grem JL, Harold N, Shapiro J, Bi DQ, Quinn MG, Zentko S, et al. Phase I and pharmacokinetic trial of weekly oral fluorouracil given with eniluracil and low-dose leucovorin to patients with solid tumors. J Clin Oncol 2000;18:3952-3963

179- Gelmon KA, Latreille J, Tolcher A, Génier L, Fischer B, Forand D, et al. Phase I dose-finding study of a new taxane, RPR 109881A, administered as a one-hour intravenous infusion day 1 and 8 to patients with advanced solid tumors. J Clin Oncol 2000;18:4098-4108

180- Patnaik A, Eckhardt SG, Izbicka E, Tolcher AA, Hammond LA, Takimoto CH, et al. A phase I, pharmacokinetic, and biological study of farnesyltransferase inhibitor tipifarnib in combination with gemcitabine in patients with adavanced malignancies. Clin Cancer Res 2003;9:4761-71

181- Gelderblom H, Salazar R, Verweij J, Pentheroudakis G, de Jonge MJA, Devlin M, et al. Phase I pharmacological and bioavailability study of oral diflomotecan (BN80915), a nove E-rig-modified campothecin analogue in adults with solid tumors. Clin Cancer Res 2003;9:4101-07

182- Murren J, Modiano M, Clairmont C, Lambert P, Savaraj N, Doyle T, et al. Phase I and pharmacokinetic study of triapine, a potent ribonucleotide reductase inhibitor, administered daily for five days in patients with advanced solid tumors. Clin Cancer Res 2003;9:4092-100

183- Mani S, McDaid H, Hamilton A, Hochster H, Cohen MB, Khabelle D, et al. Phase I clinical and pharmacokinetic study of BMS-247550, a novel derivative of epothilone B, in solid tumors. Clin Cancer Res 2004;10:1289-98

184- Mc Neel DG, Eickhoff J, Lee FT, King DM, Alberti D, Thomas JP, et al. Phase I trial of a monoclonal antibody specific for αvβ3 integrin (MEDI-522) in patients with adavanced malignancies including an assessment of the effect on tumor perfusion. Clin Cancer Res 2005;11:7851-60

185- Cunningham C, Appleman LJ, Kirvan-Visovatti M, Ryan DP, Regan E, Vukelja S, et al. Phase I and pharmacokinetic study of dolastatin-15 anlogue tasidotin (ILX651) administered intravenously on days1, 3, and 5 every 3 weeks in patients with advanced solid tumors. Clin Cancer Res 2005;11:7825-33

186- Rubin EH, de Alwis DP, Pouliquen I, Green L, Marder P, Lin Y, et al. A phase I trial of a potent P-Glycoprotein inhibitor, Zosuquidar .3HCI trihydrochloride (LY335979), administered orally in combination with doxorubicin in patients with advanced malignancies. Clin Cancer Res 2002;8:3710-17

187- Posey JA III, Saif MW, Carlisle R, Goetz A, Rizzo J, Stevenson S, et al. Phase 1study of weekly polyethylene glycol-camptothecin in patients with advanced solid tumors and lymphomas. Clin Cancer Res 2005;11:7866-71

188- Leyvraz S, Bacchi M, Cerny T, Lissoni A, Sessa C, Bressoud A, et al. Phase I multicenter study of combined high-dose ifosfamide and doxorubicin in the treatment of advanced sarcomas. Ann Oncol 1998;9:877-84

189- Rothenberg ML, Sharma A, Weiss GR, Villalona-Calero MA, Eckardt JR, Aylesworth C, et al. Phase I trial of paclitaxel and gemcitabine administered every two weeks in patients with refractory solid tumors. Ann Oncol 1998;9:733-8

190- Mross K, Hauns B, Hâring B, Bauknecht T, Meerpohl HG, Unger C, et al. Clinical phase I study with one-hour paclitaxel infusion. Ann Oncol 1998;9:569-72

191- Frasci G, Panza n, Comella P, Nicolella GP, Natale M, Pacilio C, et al. Cisplatin, gemcitabine and vinorelbine in locally advanced or metastatic non-small-cell lung cancer: A phase I study. Ann Oncol 1997;8:1045-48.

192- Ribas A, Camacho LH, Lopez-Bernstein G, Pavlov D, Bulanhagui CA, Millham r, et al. Antitumour activity in melanoma and anti-self responses in a phase I trial with the anti-cytotoxic T lymphocyte-associated antigen 4 monoclonal antibody CP-675,206. J Clin Oncol 2005;23:8968-77

193- Giles FJ, Cortes JE, Thomas DA, Garcia-Manero G, Faderl S, Jeha S, et al. Phase I and pharmacokinetic study of DX-8951f (Exatecan mesylate), a hexacyclic camptothecin, on a daily-times-five schedule in patients with advanced leukaemia. Clin Cancer Res 2002;8:2134-41

194- Cragg LH, Andreeff M, Feldman E, Roberts J, Murgo A, Winning M, et al. Phase I trial and correlative laboratory studies of bryostatin 1 (NSC 339555) and high-dose 1-B-d-Arabinofuranosylcytosine in patients with refractory acute leukaemia. Clin Cancer Res 2002;8:2123-33

195- Cohen SJ, Leichman CG, Yeslow G, Beard M, Proefrock A, Roedig B, et al. Phase I and pharmacokinetic study of once daily oral administration of S-1 in patients with advanced cancer. Clin Cancer Res 2002;8:2116-22

196- Schnell R, Staak O, Borchmann P, Schwartz C, Matthey B, Hansen H, et al. A phase I study with an anti-CD30 ricin 1-Chain immunotoxin (Ki-4.dgA) in patients with refractory CD30+ Hodgkin’s and non-Hodgkin’s lymphoma. Clin Cancer Res 2002;8:1779-86

197- Frankel AE, Powell BL, Hall PD, Case D, Kreitman RJ. Phase I of a novel diphtheria toxin/granulocyte macrophage colony-stimulating factor fusion protein (DT””8GMCSF) for refractory or relapsed acute myeloid leukaemia. Clin Cancer Res 2002;8:1004-13

198- Mani S, Rudin CM, Kunkel K, Holmlund JT, Geary RS, Kindler HL, et al. Phase I clinical and pharmacokinetic study of protein kinase C-α antisense oligonucleotide ISIS 3521 administered in combination with 5-fluorouracil and leucovorin in patients advanced cancer. Clin Cancer Res 2002;8:1042-48

199- Chang AE, Redman BG, Whitfield JR, Nickoloff BJ, Braun TM, Lee PP, et al. A phase I trial of tumor lysate-pulsed dendritic cells in the treatment of advanced cancer. Clin Cancer Res 2002;8:1021-32

200- Pitot HC, Reid JM, Sloan JA, Ames MM, Adjeij AA, Rubin J, et al. A phase I study of bizelin (NSC 615291) in patients with advanced solid tumors. Clin Cancer Res 2002;8:712-17

201- Liu G, Berlin J, Tutsch KD, Van Ummersen L, Dresen A, Marnocha R, et al. Phase I clinical and pharmacokinetic study of oral penclomedine (NSC 338720) in adults with advanced solid malignancy. Clin Cancer Res 2002;8:706-11

202- Kurtzberg J, Ernst TJ, Keating MJ, Gandhi V, Hodge JP, Kisor DF, et al. Phase I study of 506U78 administered on a consecutive 5-day schedule in children and adults with refractory hematologic malignancies. J Clin Oncol 2005;23:3396-3403

203- Mayer A, Francis RJ, Sharma SK, Tolner B, Springer CJ, Martin J, et al. A phase I study of a single administration of antibody-directed enzyme prodrug therapy with the recombinant anti-carcinoembryonic antigen antibody-enzyme fusion protein MFECP1 and a bis-iodo phenol mustard prodrug. Clin Cancer Res 2006;12:6509-16

204- Faivre S, Delbado C, Vera C, Robert C, Lozahic S, Lassau N, et al. Safety, pharmacokinetic, and antitumor activity of SU11248, a novel oral multitarget tyrosine kinase inhibitor in patients with cancer. J Clin Oncol 2006;24:24-35

205- Basche M, Gustafson DL, Holden SN, O’Bryant CL, Gore L, Witta S, et al. A phase I biological and pharmacologic study of heparanase inhibitor PI-88 in patients with advanced solid tumors. Clin Cancer Res 2006;12:5471-80

206- Roberts JD, Smith MR, Feldman EJ, Cragg L, MIllenson MM, Roboz GJ, et al. Phase I study of bryostatin 1 and fludarabine in patients with chronic lymphocytic leukaemia and indolent (non-Hodgkin’s) lymphoma. Clin Cancer Res 2006;12:5809-16

207- Schrump DS, Fischette MR, Nguyen DM, Zhao M, Li X, Kunst TF, et al. Phase I study of decitabine-mediated gene expression in patients with cancers involving the lungs, esophagus or pleura. Clin Cancer Res 2006;12:5777-85

208- Papadimitrakopoulou V, Agelaki S, Tran HT, Kies M, Gagel R, Zinner R, et al. Phase I study of the farnesyltransferase inhibitor BMS-214662 given weekly in patients with solid tumors. Clin Cancer Res 2005;11:4151-59

209- Grem JL, Morrison G, Guo XD, Agnew E, Takimoto CH, Thomas R, et al. Phase I and pharmacologic study of 17-(allylamino)-17-demethoxygeldanamycin in adult patients with solid tumors. J Clin Oncol 2005;23:1885-93.

210- Marcucci G, Stock W, Dai G, Klisovic RB, Liu S, Klisovic MI, et al. Phase I study of oblimersen sodium, an antisense to Bcl-2, in untreated older patients with acute myeloid leukemai: pharmacokinetics, pharmacodynamics, and clinical activity. J Clin Oncol 2005;23:3404-11

211- Sabbatini P, Dupont J, Aghajanian C, Derosa F, Poynor E, Anderson S, et al. Phase I study of abagovomab in patients with epithelial ovarian, fallopian tube or primary peritoneal cancer. Clin Cancer Res 2006;12:5503-10

212- Bander NH, Milowsky MI, Nanus DM, Kostakoglu L, Vallabhajosula S, Goldsmith SJ. Phase I trial of 177 lutetium-labeled J591, a monoclonal antibody to prostate-specific membrane antigen, in patients with androgen-independent prostate cancer. J Clin Oncol 2005;23:4591-601

213- Ryan QC, Headlee D, Acharya M, Sparreboom A, Trepel JB, Ye J, et al. Phase I and pharmacokinetic study of MS-275, a histone deacetylase inhibitor, in patients with advanced and refractory solid tumors or lymphoma. J Clin Oncol 2005;23:3912-22

214- Thomas AL, Morgan B, Horsfield MA, Higginson A, Kay A, Lee L, et al. Phase I study of the safety, tolerability, pharmacokinetic, and pharmacodynamics of PTK787/ZK 222584 administered twice daily in patients with advanced cancer. J Clin Oncol 2005;23:4162-71

215- Sharma RA, Euden SA, Platton SL, Cooke DN, Shafayat A, Hewitt HR, et al. Phase I clinical trial of oral curcumin : biomarkers of systemic activity and compliance. Clin Cancer Res 2004;10:6847-54

216- Cooper BW, Veal GJ, Radivoyevitch T, Tilby MJ, Meyerson HJ, Lazarus HM, et al. A phase I and pharmacodynamic study of fludarabine, carboplatin and topotecan in patients with relapsed, refractory, or high-risk leukaemia. Clin Cancer Res 2004;10:6830-39

217- Kelly WK, O’Connor OA, Krug LM, Chiao JH, Heaney M, Curey T, et al. Phase I study of an oral histone deacetylase inhibitor, suberoylanilide hydoxamic acid, in patients with adavanced cancer. J Clin Oncol 2005;23:3923-31

218- Calvo E, Tolcher AW, Hammond LA, Patnaik A, de Bono JS, Eiseman IA, et al. Administration of CI-1033, an irreversible pan-erbB tyrosine kinase inhibitor, is feasible on a 7-day on, 7-day off schedule : a phase I pharmacokinetic and food effect study. Clin Cancer Res 2004;10:7112-20

219- Hande KR, Collier M, Paradisio L, Stuart-Smith J, Dixon M, Clendeninn N, et al. Phase I and pharmacokinetic study of prinomastat, a matrix metalloprotease inhibitor. Clin Cancer Res 2004;10:909-15

220- Raymond E, Burris HA, Rowinsky EK, Eckardt JR, Rodriguez G, Smith L, et al. Phase I study of daily times five topotecan and single injection of cisplatin in patients with previously untreated non-small-cell lung carcinoma. Ann Oncol 1997:8;1003-8

221- Stevenson JP, DeMaria D, Sludden J, Kaye SB, Paz-Ares L, Grochow LB, et al. Phase I/pharmacokinetic study of topoisomerase I inihibitor GG211 administered as a 21-day continuous infusion. Ann Oncol 1999;10:339-44

222- Dimery IW, Hong WK, Lee JJ, Guillory-Perez C, Pham F, Fritsche HA, et al. Phase I trial of alpha-tocopherol effects on 13-cis-retinoic acid toxicity. Ann Oncol 1997;8:85-89

223- Budman DR, Weiselberg L, O’Mara V, Buchbinder A, Lichtman SM, Donahue L, et al. A phase I study of sequential vinorelebine followed by pacliatxel. Ann Oncol 1999;10:861-63

224- Prince HM, Millward MJ, Rischin D, Blakey D, Francis P, Gates P, et al. Repetitive high-dose therapy with ifosfamide, thiotepa and paclitaxel with peripheral blood progenitor cell and filgrastim support for metastatic and locally advanced breast cancer: results of a phase I. Ann Oncol 1999;10:479-81

225-Boyer MJ, Zalcberg J, Olver IN, Millward MJ, Richardson G, McKeage MJ. Phase I study of paclitaxel and oral etoposide in previously untreated non-small-cell and extensive small-cell lung cancer. Ann Oncol 1997;8:485-89

226- Propper DJ, Braybrooke JP, Taylor DJ, Lodi R, Styles P, Cramer JA, et al. Phase I trial of the selective mitochondrial toxin MKT 077 in chemo-resistant solid tumours. Ann Oncol 1999;10:923-27

227- Cortes J, Faderl S, Estey E, Kuzrock R, Thomas D, Beran M, et al. Phase I study of BMS-214662, a farnesyl transeferase inhibitor in atients with acute leukemias and high-risk myelodysplastic syndromes. J Clin Oncol 2005;23:2805-12

228- Frasci G, Comella P, Parziale A, Casaretti R, Daponte A, Gravina a, et al. Cisplatin-paclitaxel weekly Schedule in advanced solid tumors: a phase I study. Ann Oncol 1997;8:291-93

229- Sessa C, Minoia C, Ronchi A, Zuchetti M, Bauer J, Borner M, et al. Phase I clinical and pharmacokinetic study of the oral platinum analogue JM216 given daily for 14 days. Ann Oncol 1998;9:1315-22

230- Stupp R, Bauer J, Pagani O, Gerard B, Cerny T, Sessa c, et al. Ventricular arrhythmia and torsade de pointe: dose limiting toxicities of the MDR-modulator S9788 in a phase I. Ann Oncol 1998;9:1233-42

231- Beran M, O’Brien S, Thomas DA, Tran HT, Cortes-Franco JE, Giles F, et al. Phase I study of oral topotecan in haematological malignancies. Clin Cancer Res 2003;9:4084-91

232- Johansen M, Kuttesch J, Bleyer WA, Krailo M, Ames M, Madden T. Phase I evaluation of oral and intravenous vinrolebine in pediatric cancer patients: a report from the Children’s Oncology Group. Clin Cancer Res 2006;12:516-22

233- Kasamon YL, Flinn IW, Grever MR, Diehl LF, Garrett-Mayer E, Goodman SN, et al. Phase I study of low-dose interleukine-2, fludarbine, and cyclphosphamide for previously untreated indolent lymphoma and chronic lymphocytic leukaemia. Clin Cancer Res 2005;11:8413-17

234- Goetz MP, Toft D, Reid J, Ames M, Stensgard B, Safgren S, et al. Phase I trial of 17-allylamino-17-demethoxygeldanamycin in patients with advanced cancer. J Clin Oncol 2005;23:1078-87

235-Tijnk BM, Buter J, de Bree R, Giaccone G, Lan MS, Staab A, et al. A phase I dse escalation study with anti-CD44v6 bivatuzumab mertasine in patients with incurable squamous cell carcinoma of head and neck or esophagus. Clin Cancer Res 2006;12:6064-72

236-Khuri FR, Glisson BS, Kim ES, Statkevich P, Thall PF, Meyers ML, et al. Phase I study of the farnesyl transeferase inhibitor lonafarnib with paclitaxel in solid tumors. Clin Cancer Res 2004;10:2968-76

237- Mackay HJ, Hoekstra R, Eskens FALM, Loos WJ, Crawford D, Voi M, et al. A phase I pharmacokinetic and pharmacodynamic study of the farnesyl transferase inhibitor BMS-214662 in combination with cisplatin in patients with advanced solid tumors. Clin Cancer Res 2004;10:2336-44

238- Van Zuylen L, Bridgewater J, Sparreboom A, Eskens FALM, de Bruijn P, Sklenar I, et al. Phase I and pharmacokinetic study of the polyamine synthesis inhibitor SAM486A in combination with 5-fluorourcil/leucovorin in metastatic colorectal cancer. Clin Cancer Res 2004;10:1949-55

239- Polee MB, Sparreboom A, Eskens FALM, Hoekstra R, van de Schaaf J, Verweij j, et al. A phase I and pharmacokinetic study of weekly paclitaxel and carboplatin in patients with metastatic esophageal cancer. Clin Cancer Res 2004;10:1928-34

240- Nakata B, Mitachi Y, Tsuji A, Yamamitsu S, Hirata K, Shirasaka T, et al. Combination phase I trial of a novel oral fluorouracil derivative S-1 with low-dose cisplatin for unresectable and recurrent gastric cancer (JFMC27-9902). Clin Cancer Res 2004;10:1664-69

241- Beer TM, Garzotto M, Lowe BA, Ellis W, Montalto MA, Lange PH, et al. Phase I study of weekly mitoxantrone and docetaxel before prostatectomy in patients with high-risk localized prostate cancer. Clin Cancer Res 2004;10:1306-11

242- Briasoulis A, Judson I, Pavlidis N, Beale P, Wanders J, Groot Y, et al. Phase I trial of 6-hour infusion of glufosfamide, a new alkylating agent with potentially enhanced selectivity for tumors that overexpress transmembrane glucose transporters: a study of the European Organization for Research and Treatment of Cancer Early Clinical Studies Group. J Clin 2000;18:3535-44

243- Seiden V, Ng SW, Supko JG, Ryan DP, Clark JW, Lynch T, et al. A phase I clinical trial of sequentially administered doxorubicin and topotecan in refractory solid tumors. Clin Cancer Res 2002;8:691-97

244- Daw NC, Santan VM, Iacono LC, Furman WL, Hawkins DR, Hoghton PJ, et al. Phase I and pharmacokinetic study of topotecan administered orally once daily for 5 days for 2 consecutive weeks to pediatric patients with refractory solid tumors. J Clin Oncol 2004;22:829-37

245- Thompson JA, Curti DB, Redman BG, Bhatia S, Weber JS, Argawala SS, et al. Phase I study of recombinant interleukin-21 in patients with metastatic melanoma and renal cell carcinoma. J Clin Oncol 2008;12:2034-39

246- Drevs J, Siegert P, Medinger M, Mross K, Strecker R, Zirrgiebel U, et al. Phase I clinical study if AZD2171, an oral vascular endthelial growth factor signaling inhibitor, in patients with advanced solid tumors. J Clin Oncol 2007;25:3405-54

247- Rudin CM, Kozloff M, Hoffman PC, Edelman MJ, Karnauskas R, et al. Phase I study of G319, a bcl-2 antisense oligonucleotide, combined with carboplatin and etoposide in patients with small-cell lung cancer. J Clin Oncol 2004;22:1110-17

248- O’Donnell A, Faivre S, Burris HA III, Rea D, Papadimitrakopoulou V, Shand n, et al. Phase I pharmacokinetic and pharmacodynamic study of the oral mammalian target of rapamycin inhibitor everolimus in patients with advanced solid tumors. J Clin Oncol 2008;26:1588-95

249- Gandhi V, Tam C, O’Brien S, Jewell RC, O’Rodriguez C, Lerner S, et al. Phase I trial of nelarabine in indolent leukemias. J Cln Oncol 2008;26:1098-1105

250- Schwartz G, O’Reilly E, Ilson D, Saltz L, Sharma S, Tong W, et al. Phase I study of the cyclin-dependent kinase inhibitor flavopiridol in combination with paclitaxel in patients with adavanced solid tumors. J Clin Oncol 2002;20:2157-70

251- Schwartz GK, Weitzman A, O’Reilly E, Brail L, de Alwis DP, Cleverly A, et al. Phase I and pharmacokinetic study of LY293111, an orally bioavailable LTB4 receptor antagonist, in patients with advanced tumors. J Clin Oncol 2006;23:5365-73

252- Burris HA III, Hurwitz HI, Dees EC, Dowlati A, Blackwell KL, O’Neil B, et al. Phase I safety, pharmacokinetics, and clinical activity study of lapatinib (GW572016), a reversible dual inhibitor of epidermal growth factor receptor tyrosine kinases, in heavily pretreated patients with metastatic carcinoma. J Clin Oncol 2005;23:5305-13

253- Jayson GC, Mulatero C, Ranson M, Zweit J, Jackson A, Broughton J, et al. Phase I investigation of recombinant anti-human vascular endothelial growth factor antibody in patients with advanced cancer. Eur J Cancer 2005;41:555-63

254- Rustin GJS, Galbraith SM, Anderson H, Stratford M, Folkes LK, Sena L, et al. Phase I clinical trial of weekly combrestatin A4 phosphate: clinical and pharmacokinetic results. J Clin Oncol 2003;2&:2815-22

255- Abraham J, Agrawal M, Bakke S, Rutt a, Edgerl M, Balis FM, et al. Phase I trial and pharmacokinetic study of BMS-247550, an epothilone B analog, administered intravenously on a daily schedule for five days. J Clin Oncol 2003;21:1866-73

256- Sawyer MB, Ratain MJ, Bertucci D, Smith RP, Schilsky RL, Vogelzang NJ, et al. Phase I study of an oral formulation of ZD9331 administered daily for 28 days. J Clin Oncol 2003;2&:1859-65

257- Kantarjian H, Gandhi V, Kozuch P, Faderl S, Giles F, Cortes J, et al. Phase I clinical and pharmacology study of clorafabine in patients with solid and hematologic cancers. J Clin Oncol 2003;21:1167-73

258- Chester JD, Joel SP, Cheeseman SL, Hall GD, Braun MS, Perry J, et al. Phase I and pharmacokinetic study of intravenous irinotecan plus oral ciclosporin in patients with fluorouracil-refractory colon cancer. J Clin Oncol 2003;21:1125-32

259- Pecora AL, Rizi N, Cohen GI, Meropol NJ, Sterman D, Marshall JL, et al. Phase I trial of intravenous administration of PV701, an oncolytic virus, in patients with advanced solid cancers. J Clin Oncol 2002;20:2251-66

260- Beerepoot LV, Radema SA, Witteveen EO, Thomas T, Wheeler C, Kempin S, et al. Phase I clinical evaluation of weekly administration of the novel vascular-targeting agent, ZD6126, in patients with solid tumors. J Clin Oncol 2006;24:1491-98

261- Tolcher AW, Hao D, de Bono J, Miller A, Patnaik A, Hammond LA, et al. Phase I, pharmacokinetic, and pharmacodynamic study of intravenously administered Ad5CMV-p53, an adenoviral vector containing wild-type p53 gene, in patients with advanced cancer. J Clin Oncol 2006;24:2052-58

Prend en compte les DLT de tous les cycles

262- Hudes G, Haas N, Yeslow G, Gillon T, Gunnarsson PO, Ellman M, et al. Phase I clinical and pharmacologic trial of intravenous estramustine phosphate. J Clin Oncol 2002;20:1115-27

263- Kehrer DFS, Bos AM, Verweij J, Groen HJ, Loos WJ, Sparreboom A, et al. Phase I and pharmacologic study of liposomal lutrotecan, NX 211: urinary excretion predicts hematologic toxicity. J Clin Oncol 2002;20:1222-31

264- Sparano JA, Speyer J, Gradishar WJ, Liebes L, Sridhara R, Mendoza S, et al. Phase I trial of escalating doses of paclitaxel plus doxorubicin and dexrazone in patients with advanced breast cancer. J Clin Oncol 1999;17:880-86

265- Blakely LJ, Budzar A, Chang HY, Frye D, Theriault R, Valero V, et al. A phase I and pharmacokinetic study of TAS-108 in postmenopausal female patients with locally advanced, locally recurrent inoperable, or progressive metastatic breast cancer. Clin Cancer Res 2004;10:5425-31

266- Bélanger K, Moore M, Baker SD, Dionne J, Maclean M, Jolivet J, et al. Phase I and pharmacokinetic study of novel l-nucleoside analog troxacitabine given as a 30-minute infusion every 21 days. J Clin Oncol 2002;20:2567-74

267- Furman WL, Crews KR, Billups C, Wu J, Gajjar AJ, Daw NC, et al. Cefixime allows greater dose escalation of oral irinotecan: a phase I study in pediatric patients with refractory solid tumors. J Clin Oncol 2006;24:563-70

268- Munster P, Marchion D, Bicaku E, Schmitt M, Lee JH, DeConti R, et al. Phase I trial of histone deacetylase inhibition by valproic acid followed by the topoisomerase II inhibitor epirubicin in advanced solid tumors: a clinical and translational study. J Clin Oncol 2007;25:1979-85

269- Villablanca JG, Krailo MD, Ames MM, Reid JM, Reaman GH, Reynolds CP. Phase I trial of oral fentertinide in children with high-risk solid timors: a report from the Children’s Oncology Group (CCG 09709). J Clin Oncol 2006;24:3423-30

270- Goh BC, Ratain MJ, Bertucci D, Smith R, Mani S, Vogelzang NJ, et al. Phase I study of ZD9331 on short daily intravenous bolus infusion for 5 days every 3 weeks with fixed dosing recommendations. J Clin Oncol 2001;19:1476-84

271- Bender JLG, Adamson PC, Reid JM, Xu L, Baruchel S, Shaked Y, et al. Phase I trial and pharmacokinetic study of bevacizumab in pediatric patients with refractory solid tumors : a Children’s Oncology Group Study. J Clin Oncol 2008;26:399-405

272- El-Rayes B, Gadgeel S, Shields AF, Manza S, Lorusso P, Philip PA. Phase I study of bryostatin and gemcitabine. Clin Cancer Res 2006;12:7059-62

273- Madajewicz S, Hentschel P, Burns P, Caruso R, Fiore J, Fried M, et al. Phase I chemotherapy study of biochemical modulation of folinic acid and fluorouracil by gemcitabine in patients with solid tumors.malignancies. J Clin Oncol 2000;18:3553-57

274- Vanderheide RH, Dutcher JP, Anderson JE, Eckhardt SG, Stephans KF, Razvillas B, et al. Phase I study of recombinant human CD40 ligand in cancer patients. J Clin Oncol 2001;19:3280-87

275- Seymour LW, Ferry DR, Anderson D, Hesslewood S, Julyan PJ, Payner R, et al. Hepatic drug targeting: phase evaluation of polymer-bound doxorubicin. J Clin Oncol 2002;20:1668-76

276- Garrison MA, Hammond LA, Geyer CE, Schwartz G, Tolcher AW, Smetzer L, et al. A phase I and pharmacokinetic study of exatecan mesylate administered as a protracted 21-day infusion in patients with advanced malignancies. Clin Cancer Res 2003;9:2527-37

277- Hoff PM, Saad ED, Ajani JA, Lassere Y, Wenske C, Medgyesy D, et al. Phase I study with paharmacokinetics of S-1 on an oral daily schedule for 28 days in patirnts with solid tumors. Clin Cancer Res 2003;9:134-142

278- Witta SE, Gustafson DI, Pierson AS, Menter A, Holden SN, Basche M, et al. A phase I and pharmacokinetic study of exisulind and docetaxel in patients with advanced solid tumors. Clin Cancer Res 2004;10:7279-37

279- Rudin CM, Marshall JL, Huang CH, Kindler HL, Zhang C, Kumar D, et al. Delivery of a liposomal c-raf-1 antisense oligonucleotide by weekly bolus dosing in patients with advanced solid tumors: a phase I. Clin Cancer Res 2004;10:7244-51

280- Garrett CR, Fishman MN, Rago RR, Williams CC, Dellaportas AM, Mahany JJ, et al. Phase I study of a novel taxane BMS-188797 in adult patients with solid malignancies. Clin Cancer Res 2005;11:3335-41

281- de Jonge MJA, van der Gaast A, Planting AST, Van Doorn L, Lems A, Boot I, et al. Phase I and pharmacokinetic study of the dolastatin 10 analogue TZT-1027, givenon days 1 and 8 of a 3-week cycle in patients with advanced solid tumors. Clin Cancer Res 2005;11:3806-13

282- Lokich J. Phase I clinical trial of weekly combined topotecan and irinotecan. Am J Clin Oncol 2001;24:336-40

283- ten Tije AJ, Verweij J, Sparreboom A, van der gaast A, Fowst C, Fiorentini F, et al. Phase I and pharmacokinetic study of brostallicin PNU-166196), a new DNA minor-groove binder, administered intravenously every 3 weeks to adult patients with metastatic cancer. Clin Cancer Res 2003;9:2957-64

285- Zonnenberg BA, Groenewegen G, Janus TJ, Leahy TW, Humerickhouse RA, Isaacson JD, et al. Phase I dose-escalation study of the safety and pharmacokinetics of atrasentran: an endothelin receptor antagonist for refractory prostate cancer. Clin Cancer Res 2003;9:2965-72

286- deJonge MJA, Sparreboom A, Planting AST, Van der Burg MEL, de Boer-Dennert MM, Steeg JT, et al. Phase I study of 3-weeks schedule of irinotecan combined with cisplatin in patients with advanced solid tumors. J Clin Oncol 2000;18:187-94

287- Dodd PM, McCaffrey JA, Hilton S, Mazumdar M, Herr H, Kelly WK, Icasiano E, et al. Phase I evaluation of sequential doxorubicin+gemcitabine then ifosfamide+ paclitaxel + cisplatin for patients with unresectable or metastatic transitional-cell carcinoma of the urothelial tract. J Clin oncol 2000;18:840-46

288- Wolff AC, Donehower RC, Carducci MK, Carducci MA, Brahmer JR, Zabelina Y, et al. Phase I study of docosahexaenoic acid-paclitaxel: a taxane-fatty acid conjugate with a unique pharmacology and toxicity profile. Clin Cancer Res 2003;9:3589-97

289- Erlichman C, Hidalgo M, Boni J, Martins P, Quinn S, Zacharchuk C, et al. Phase I study of EKB-569, an irreversible inhibitor of the epidermal growth factor receptor, in patients with advanced solid tumors. J Clin Oncol 2006;24:2252-60

290- Milowsky MI, Nanus DM, Kostakoglu L, Vallabhajosula S, Goldsmith SJ, Bander NH. Phase I trial of Yttrium-90-labelled anti-prostate-specific membrane antigen monoclonal antibody J91 for androgen-independent prostate cancer. J Clin Oncol 2004;22:2522-31

291- Shah MA, Kortmansky J, Motwani M, Drobnjak M, Gonen M, Yi S, et al. A phase I clinical trial of the sequential combination of irinotecan followed by flavopiridol. Clin Cancer Res 2005;11:3836-45

292- Jimeno A, Li J, Messersmith WA, Rudeck MA, Maniar M, Hidalgo M, Baker SD, et al. Phase I of ON 01910.Na, a novel modulator of the polo-like kinase 1 pathway, in adult patients with solid tumors. J Clin Oncol 2008;26:5504-10.

293- MacDonald TJ, Stewart CF, Kocak M, Goldman s, Ellenbogen RG, Philips P, et al. Phase I clinical trial of cilengitide in children with refractory brain tumors: pediatric brain tumor consortium study PBTC-012. J Clin Oncol 2008;26:919-24

294- Goldwasser F, Gross-Goupil M, Tigaud JM, Di Palma M, Marceau-Suissa J, Wasserman E, et al. Dose escalation of CPT-11 in combination with oxaliplatin using an every two weeks schedule : A phase I study in advanced gastrointestinal cancer patients. Ann Oncol 2000;11:1463-70

295- Gillespie AM, Broadhead TJ, Chan SY, Owen J, Farnsworth AP, Sopwith M, et al. Phase I pen study of the effects of ascending doses of the cytotoxic immunoconjugate CMB-401 (hCTMO1-calicheamicin) in patients with epithelial ovarian cancer. Ann Oncol 2000;11:735-41

296- Bonneterre J, Chevalier B, Focan C, Mauriac L, Piccart M. Phase I and pharmacokinetic study of weekly oral therapy with vinorelabine in patents with advanced breast cancer (ABC). Ann oncol 2001;12:1683-91

297- Villano-Calero MA, Eder JP, Toppmeyer DL, Allen LF, Fram R, Velagapudi R, et al. Phase I and pharmacokinetic study of LU79553, a DNA intercalating bisnaphthalimide, in patients wih solid malignancies. J Clin Oncol 2001;19:857-69.

298- Zujewski J, Horak ID, Bol CJ, Woestenborghs R, Bowden C, End DW, et al. Phase I and pharmacokinetic study of farnesyl protein transferase inhibitor R115777 in advanced cancer. J Clin Oncol 2000;18:927-41

299- Motzer RJ, Rakhit A, Ginsberg M, Rittweger K, Vuky J, Yu R, et al. Phase-I trial of 40-kd branched pegylated interferon alfa-2a for patients with advanced renal cell carcinoma. J Clin Oncol 2001;19:1312-19

300- Petrylak DP, MacArthur RB, John O’Connor, Shelton G, Judge T, Balog J, et al. Phase I trial of docetaxel with estramustine in androgen-independent prostate cancer. J Clin Oncol 1999;17:98-67

301- Belani CP, Kearns CM, Zuhowski EG, Erkmen K, Hiponia D, Zacharski D, et al. Phase I trial, including pharmacokinetic and pharmacodynamic correlations, of combination paclitaxel and carboplatin in patients with metastatic non-small-cell lung cancer. J Clin Oncol 1999;17:676-84

302- Barneji U, O’Donnell A, Scurr M, Pacey M, Stapleton S, Asad Y, et al. Phase I pharmacokinetic and pharmacodynamic study of 17-allylamino, 17-demethoxygeldanamycin in patients with advanced malignancies. J Clin Oncol 2005;23:4152-61

303- Villalona-Calero MA, Wientjes MG, Otterson GA, Kanter S, Young D, Murgo AJ, et al. Phase I study of low-dose suramin as a chemosensitizer in patients with advanced non-small cell lung cancer. Clin Cancer Res 2003;9:3303-3311

304- Takimoto CH, Morrison G, Harold N, Quinn M, Monahan BP, Band RA, et al. Phase I and pharmacologic study of irinotecan administered as a 96-hour infusion weekly to adult cancer patients. J Clin Oncol 2000;18:659-67

305- de Jonge MJA, Punt CJA, Gelderblom H, Loos WJ, Van Beurden V, Planting AST, et al. Phase I and pharmacologic study of oral (PEG-1000) 9-aminocamptothecin in adult patients with solid tumors. J Clin Oncol 1999;17:2219-26

306- Schey SA, Fields P, Bartlett JB, Clarke LA, Ashan G, Knight RD, et al. Phase I study of an immunomodulatory thalidomide analog, CC-4047, in relapsed or refractory myeloma. J Clin Oncol 2004;22:3269-76

307- Reid JM, Qu W, Safgren SL, Ames MM, Krailo MD, Seibel NL, et al. Phase I trial and pharmacokinetics of gemcitabine in children with advanced solid tumors. J Clin Oncol 2004;22:2445-51

308- Gilbert MR, Supko JG, Batchelor T, Lesser G, Fischer JD, Piantadosi S, et al. Phase I pharmacokinetic and pharmacodynamic study of weekly 1-hour and 24-hour infusion BMS-214662, a farnesyltransferase inhibitor, in patints with advabced solid tumors. J Clin Oncol 2005;23:2521-33

309- Taberno J, Rojo F, Marimon I, Voi M, Albanell J, Guix M, Vazquez F, et al. Phase I pharmacokinetic and pharmcodynamic study of weekly 1-hour and 24-hour infusion BMS-214662, a farnesyltransferase inhibitor, in patients with advanced solid tumors. J Clin Oncol 2005;23:2521-33

310- Hoekstra R, Dumez H, Eskens FAL, Van der Gaast A, Planting AST, de Heus G, et al. Phase I and pharmacologic study of PKI166, an epidermal growth factor receptor tyrosine kinase inhibitor, in patients with advanced solid malignancies. Clin Cancer Res 2005;11:6908-14

311-Rowinsky EK, Humphrey R, Hammond LA, Aylesworth C, Smetzer L, Hidalgo M, et al. Phase I and pharmacologic study of the specific matrix metalloprotease inhibitor BAY-9566 on a protracted oral daily dosing schedule in patients with solid malignancies. J Clin Oncol 2000;18:178-186
